# Beyond ManifoldEM: geometric relationships between manifold embeddings of a continuum of 3D molecular structures and their 2D projections†

**DOI:** 10.1039/d2dd00128d

**Published:** 2023-04-06

**Authors:** Evan Seitz, Joachim Frank, Peter Schwander

**Affiliations:** a Department of Biochemistry and Molecular Biophysics, Columbia University Medical Center New York NY 10032 USA ees2192@columbia.edu jf2192@cumc.columbia.edu; b Department of Biological Sciences, Columbia University New York NY 10027 USA; c Department of Physics, University of Wisconsin-Milwaukee Milwaukee WI 53211 USA pschwan@uwm.edu

## Abstract

ManifoldEM is an established method of geometric machine learning developed to extract information on conformational motions of molecules from their projections obtained by cryogenic electron microscopy (cryo-EM). In a previous work, in-depth analysis of the properties of manifolds obtained for simulated ground-truth data from molecules exhibiting domain motions has led to improvements of this method, as demonstrated in selected applications of single-particle cryo-EM. In the present work this analysis has been extended to investigate the properties of manifolds constructed by embedding data from synthetic models represented by atomic coordinates in motion, or three-dimensional density maps from biophysical experiments other than single-particle cryo-EM, with extensions to cryo-electron tomography and single-particle imaging with a X-ray free-electron laser. Our theoretical analysis revealed interesting relationships between all these manifolds, which can be exploited in future work.

## Abstract 2

This study explores the geometric relationships between manifolds of molecular conformers obtained from different types of experimental data.

## Graphical abstract

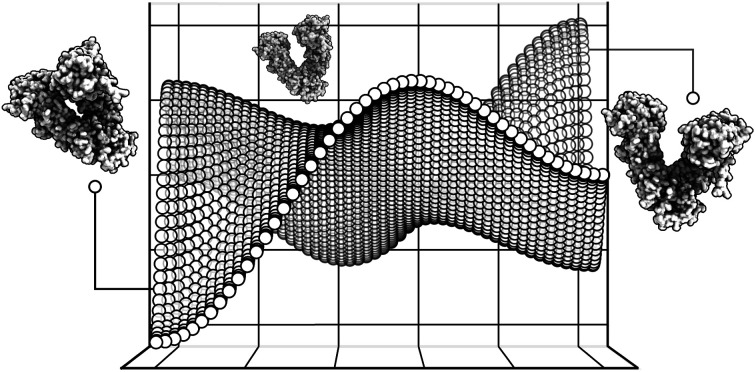

## 1 Introduction

Biological molecules and their assemblies, including molecular machines, assume a continuum of conformational states as they go through work cycles required for executing their metabolic function.^1,2^ Single-particle cryo-EM^3^ of suitable *in vitro* systems affords the ability to collect a large number of projections that originate from an ensemble of 3D structures presenting a continuum of states in thermal equilibrium.^4–8^ This information, however, comes buried among typically hundreds of thousands of unorganized snapshot images, each a 2D projection of a molecular instance, corrupted by aberrations of the microscope and noise that often exceeds the signal by more than an order of magnitude. By virtue of machine learning algorithms, it is possible to determine a low-dimensional representation of the conformational spectrum, with leading coordinates corresponding to each of the system's degrees of freedom.

From the numbers of sightings of the observed states in this low-dimensional space, a free-energy landscape may be obtained following a fundamental relationship of statistical mechanics.^4,6,9^ Together this provides a complete mapping of the system's state space while articulating its energetics topographically in the form of sprawling hills and valleys.^10,11^ From such a landscape, a minimum-energy path can be derived representing the most probable sequence of transitions taken by the molecular machine between any two states.^12,13^ And along this path, a sequence of 3D structures can be extracted for biophysical analysis, allowing the basis for molecular function to be elucidated. The ability to experimentally determine energy landscapes, in conjunction with 3D conformational movies of a molecular machine along distinct low-energy paths, opens a new horizon in structural biology and, by extension, in molecular medicine.

In general, obtaining this desired information is a difficult task, and numerous methods have been developed over the last decade to tackle this problem.^5,14–16^ The ManifoldEM method^5,17^ we have adopted is based on an unsupervised geometric machine learning approach using manifold embedding to recover the distribution and occupancies‡‡We use the term “occupancy” throughout the manuscript, which corresponds to the number of states in conformational space. It is related to the conformational density of states *ρ*(*C*), so that the number of states in a small interval [*C* ± Δ*C*] is proportional to *ρ*(*C*) × Δ*C*. of states. Its viability has been demonstrated in its application to four experimental systems—the ribosome,^5^ ryanodine receptor (RyR1),^17^ vacuolar ATPase,^18^ and SARS-CoV-2 spike protein.^19^ In each of these studies, conformational states of the respective macromolecular complex were characterized by different spatial constellations of its relatively rigid domains, and organized in a state space according to the continuous motions of each domain along a unique coordinate.

Recently, we devised a protocol using ensembles of simulated macromolecular complexes to interpret the conformational manifolds learned by two prominent dimensionality-reduction methods: principal component analysis^20^ (PCA) and diffusion maps^21^ (DM, used in ManifoldEM), with a strong focus on the latter. With the knowledge gained from this analysis, we were able to introduce several advancements to the ManifoldEM approach in a novel method called ESPER (Embedded Subspace Partitioning and Eigenfunction Realignment), which we validated using experimental data of the ribosome and RyR1 obtained *via* cryo-EM.^22^ Altogether, ESPER constitutes a substantial improvement of the original ManifoldEM framework that advances the ability to accurately and efficiently construct the free-energy landscape of macromolecular complexes from cryo-EM data.^22^ Our *post hoc* interpretation strategy, however, was only briefly summarized in that work.

The current article brings our interpretation strategy center stage to detail the analysis that proved instrumental in edifying our understanding of these complex manifold geometries. In contrast to the scope of our companion article,^22^ which deals solely with the properties of 2D projections of macromolecular conformers, we also now focus on their 3D structures represented either by electron density maps or sets of atomic-coordinate structures. Ultimately, we embark on an in-depth analysis of different types of synthetic-continuum datasets and their embeddings, with each “datatype” modeled so as to emulate the output of a different experiment from the same conformations of a molecular machine.

Importantly, the insights gained from our analysis of these datatypes are not limited to the field of cryo-EM, but also apply to other biophysical methods supplying information on conformational continua. In order to put the properties of these datatypes into the context of established theories, we further frame our results in comparison to a simple approximative mathematical model and derive useful explicit relationships. In the process, many striking similarities emerge between the results from various datatypes, and when differences do emerge, the information is revelatory.

Through this analysis, we are able to demonstrate the effect of different choices of continuum datatypes on the performance of geometric machine learning, which has significant implications for the reconstruction of complex information obtained from a range of widely used experimental methods. Given these results, we are able to highlight the power of manifold embedding in elucidating important biophysical properties—provided that minimum requirements are met in the data—while describing several key challenges and limitations that must be considered when analyzing datasets with multiple types of statistical uncertainties. Finally, informed by these implications, we provide a general outlook on the future of these methods within the scope of cryo-EM and beyond.

### 1.1. Additional background

As described in our companion work,^22^ we first devised a protocol for generating ensembles of simulated cryo-EM images derived from an atomic-coordinate structure (ACS) of a known protein. Specifically, we chose the heat-shock protein Hsp90 due to its simple design, exhibiting two arm-like domains (chain A and B) connected in an overarching V-shape which are known to naturally undergo large conformational changes.^23^ We altered the 3D atomic coordinates of this molecule in its closed state (PDB 2CG9)^24^ so as to exercise multiple independent easily-identifiable conformational motions§§In the literature, there is a wide range of nomenclature used here among fields and, in some instances, works by the same authors. For clarity, the following terms are interchangeable: conformational motions (CMs); conformational coordinates (CCs); reaction coordinates (RCs); collective motion coordinates. (CMs¶¶A tabulated description of symbols and abbreviations used throughout this document is available in ESI.†) with *n* = 1, 2 or 3 degrees of freedom. As one example, to simulate the motions for *n* = 2, we independently altered the positions of the molecule's two large arm-like domains using equispaced rotations of each domain about its hinge–residue axis, which we refer to as CM_1_ and CM_2_, respectively. By exercising these domain motions independently in all combinations, we generated a set of *M*_2_ = 400 structures—each represented by its atomic coordinates in PDB format—altogether spanning a 20 × 20 state space (SS_2_, with intrinsic dimensionality *n* = 2).

These *M_n_* structures were then transformed into simulated 3D electron density||||In reality, cryo-EM data (under the regime of weak phase contrast in bright-field transmission electron microscopy) are projections of the Coulomb potential distribution, which is distinct from the electron density distribution determined in X-ray crystallography. For the present analysis, however, this distinction is irrelevant. maps (EDM) with a resolution of 3 Å, where each atom is represented by a 3D Gaussian with radius defined by the corresponding number of electrons.^25^ Using parallel line integrals along the direction of the electron beam, 2D projections of each EDM were obtained to simulate weak-phase contrast images as generated in a transmission electron microscope (TEM) operated in bright-field mode.^3^ These projections were generated for evenly-spaced viewing angles across the entire angular space, so that a full set of images depicting *M_n_* conformational states of the molecule was available for each projection direction (PD). Each projection was further modified by application of the contrast transfer function (CTF), duplication to create multiple sightings per conformational state, and addition of noise to simulate realistic images of a cryo-EM experiment.

Selected ensembles of such simulated single-particle cryo-EM images were then independently embedded in a low-dimensional space—forming a manifold of intrinsic dimension *n*—and the resulting spectral properties^26^ of each embedding were analyzed and compared. Using first pristine (*i.e.*, noiseless and aberration-free) synthetic data, we observed surprising patterns in the geometries of these embeddings. For instance, each PD-manifold followed a clearly-defined high-dimensional parabolic surface, but also included eigenfunction harmonics and other unexpected aberrations. The same geometric features were present for datasets with additive noise applied to the images, with the fidelity of these features to the pristine case dependent on the signal-to-noise ratio (SNR), the number of times *τ* images were generated for each state *M_n_* (totaling *N_n_* = *τM_n_*), and introduction of CTF.

But it was only through a much deeper investigation of the manifolds of atomic-coordinate structures and 3D electron density maps of the same conformer—which we aim to present in this article—that a complete understanding of these manifolds was obtained. Using this knowledge, we were able to determine the exact form of the eigenfunctions in each PD-manifold, including identification of each set of harmonics and high-dimensional manifold rotations.

Guided by results of these ground-truth studies on both pristine and noisy data encompassing up to three degrees of freedom, the compendium of our heuristic findings provided new insights into the origin of longstanding ManifoldEM problems, leading to the development of the ESPER method for correcting them. Along with several novel operations and refinements to the preexisting approach, the ESPER method provides a thorough rationale for properly handling the *n*-dimensional PD-manifold embeddings in the presence of experimentally-relevant noise, CTF and several degrees of freedom to accurately generate the free-energy landscape of a molecular machine as well as 3D movies depicting its function.

## 2 Relationships between manifolds formed for different representations of continuum data

In this section, we expose our findings on ACS, EDM and PD continuum datasets in detail. As the analysis leading to these findings is rooted in established spectral theory,^26–28^ we encourage readers to peruse the ESI Section A,† where we detail both the PCA and DM approach, and for the latter, introduce the Laplace-Beltrami Operator (LBO) and define key parameters such as the Gaussian bandwidth *ε*. Briefly, we note that the DM eigenvectors Ψ converge to the eigenfunctions *ψ* of the LBO on a manifold Ω, sampled at the given data points, and carry useful information about the intrinsic geometry of Ω.

**Fig. 1:**
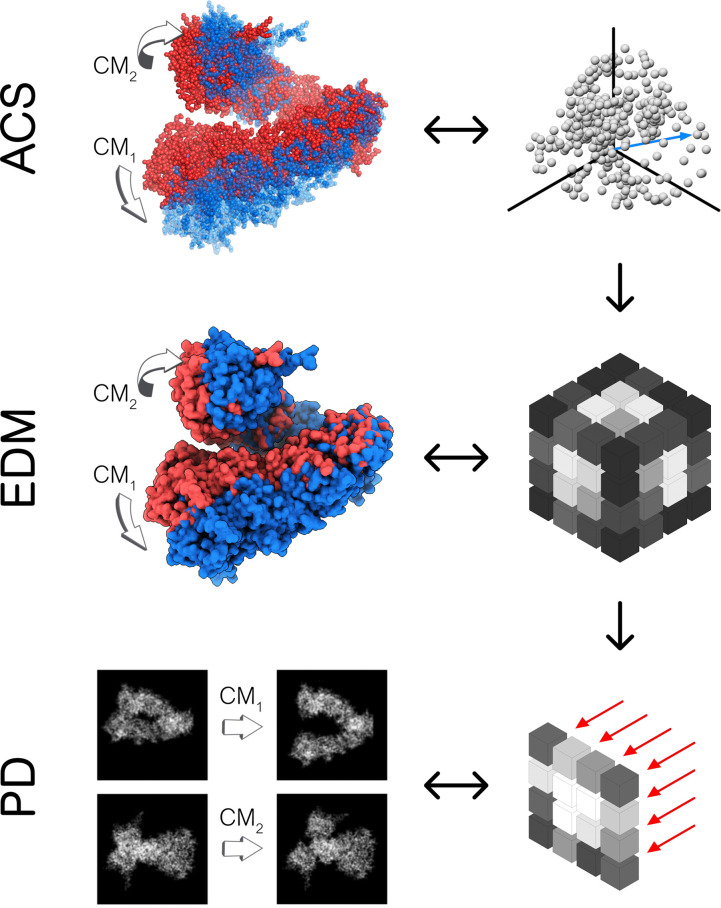
Outline of our framework for generating continuum data composed in three distinct datatypes: 3D atomic-coordinate structures (top); 3D electron density maps (middle); and 2D projections of 3D electron density maps (bottom). In the left-hand column are the respective appearance of these datatypes for the Hsp90 continuum models, showing the type and general range of motion for two conformational motions (CM_1_ and CM_2_). On the right is a corresponding schematic, showing how the continuum models are represented by the respective datatype. Specifically, a collection of 3D coordinates (top) defines the position of each atom, while a set of 3D voxels (middle)—or pixels (bottom)—defines the electron density|| within each cubic—or its projection in a square—region in space (to note, our schematic showing the projection operation along a principal coordinate is strictly for conceptual aid; in application, projections occur along any direction).

As an outline of our exposition, in Section 2.1, we use the DM method to investigate the known eigenfunctions of the LBO on the interval and rectangular domains and compare these results to the manifolds formed by a quasi-continuum of atomic-coordinate structures. Following this analysis, we detail how the structure of manifolds Ω obtained from a conformational state space transforms as the data type is changed successively from atomic-coordinate structures (Ω_ACS_) in Section 2.2, to 3D density maps (Ω_EDM_) in Section 2.3, and finally to 2D projections (Ω_PD_) of those maps in Section 2.4. A schematic of this framework is provided in Fig. 1, showing how the Hsp90 ground-truth data are innately represented by each of these distinct datatypes.

### 2.1. Eigenfunctions of the latent space

We first introduce the simplest possible continuum model, which we construct using a spatially delimited set of points X_*n*_ in 
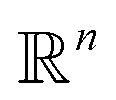
, forming a metric space with Euclidean metric^29^ defined by the equation

1 *d*(**a**, **b**) = ‖**a** − **b**‖ = [(*a*_1_ − *b*_1_)^2^ + ⋯ (*a*_*n*_ − *b*_*n*_)^2^]^1/2^

which gives the “standard” distance between any two vectors ***a*** and ***b*** in Euclidean *n*-space. Specifically, we define X_*n*_ as a Cartesian product of equispaced points along each linear dimension: for example, a line of points for *n* = 1, or an array of points arranged inside a rectangle for *n* = 2. Altogether, we term these elements and their relations the latent *n*-space.

At first glance, such a latent space seems to have little relation to the relatively complex synthetic continuum dataset of Hsp90 conformers. However, this abstraction is motivated by the representation of our ground-truth state space of atomic models, where the relationship between equispaced coordinates in the latent space matches the relationship between equiangular molecular-domain rotations. By embedding the data of the atomic-coordinate structures of Hsp90 conformers occupying SS_*n*_ and comparing the resulting eigenvectors to those obtained from the embedding of the data of the latent space X_*n*_, we will show in Section 2.2 that the results are nearly identical. We will then demonstrate in Section 2.3 and 2.4 that for the embeddings of data from the 3D electron density maps as well as their 2D projections, the relation relative to the latent space becomes distorted, which can be explained by a change of the metric in the process of switching from one data type to the other.

#### 2.1.1 State space 1

First, we will show that in the 1D space X_1_, a set of pairwise distances between a collection of equispaced coordinates on a line carries all essential information necessary to model the pairwise distances between a sequence of atomic models in SS_1_ with a molecular domain rotated by a constant angular increment. To represent our SS_1_ dataset, we uniformly sample *N* = 50 equispaced points from a 1D interval 
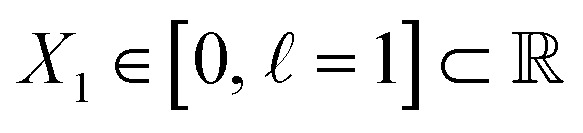
, with each of these points representing a unique state of the molecule. Following the DM method, we then calculate the distance matrix for this collection of points and embed the data in a low-dimensional space, spanned by the leading eigenvectors. Furthermore, we will show that two characteristic regimes emerge depending on the choice of Gaussian bandwidth, which we will denote with *ε*_↓_ and *ε*_↑_ for the small and large regime, respectively.

**Fig. 2:**
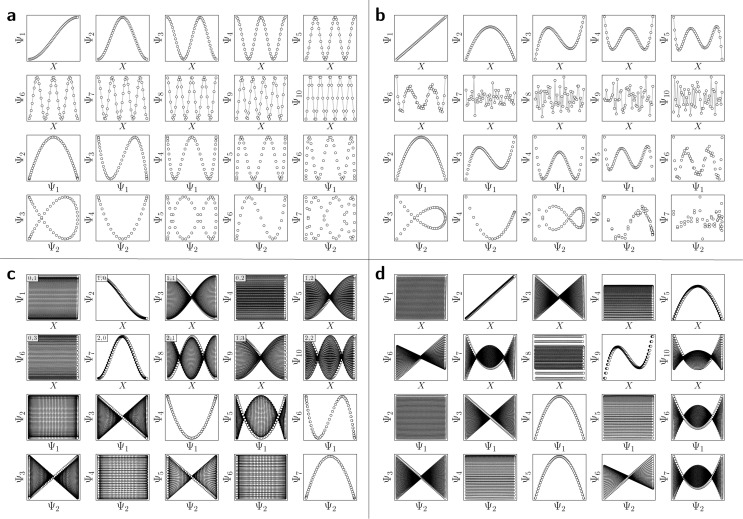
DM eigenvectors in the nondegenerate latent 1-space and 2-space. The DM eigenvectors of the 1D interval for small (*ε*_↓_ = 5 × 10^−5^) and large (*ε*_↑_ = 10) Gaussian bandwidths are shown in (a) and (b), respectively. Likewise, eigenvectors of the *N* = 50 × 50 rectangular (nondegenerate) domain for small and large Gaussian bandwidth are shown in (c) and (d), respectively. As will be done throughout this text, eigenfunctions have been independently displayed by indexing each by its ground-truth ordering (here *via* sequential *x*-coordinates). For (c) and (d), a similar appearance of eigenfunction plots, albeit interchanged, would be seen when indexing instead *via* sequential *y*-coordinates. In (c), each eigenfunction's corresponding modes {*v*,*w*} have also been provided in the top left-hand corner. For all four subplots, pairwise combinations of eigenfunctions are additionally shown, which can be visualized after an embedding without any ground-truth knowledge.

For the regime of small Gaussian bandwidths, a cosine series emerged for all eigenvectors (Fig. 2A), in very good agreement with the Laplacian eigenfunctions on a 1D Euclidean interval with Neumann boundary conditions. Specifically, we anticipate and retrieve canonical eigenfunctions^28^ of the form 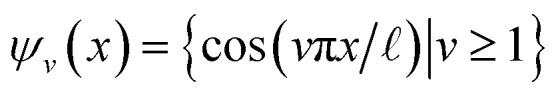. As the Gaussian bandwidth was incrementally increased from *ε*_↓_ to *ε*_↑_, this cosine series smoothly transformed into a different complete, orthogonal set: the Legendre polynomials^30^ (Fig. 2B). However, we note that these polynomials only occur for boundary conditions of hyperrectangles, which are *n*-dimensional Cartesian products of orthogonal intervals.

#### 2.1.2 State space 2

Next, to represent our SS_2_ dataset, we uniformly sample *N* = 50 × 50 points from a 2D interval 
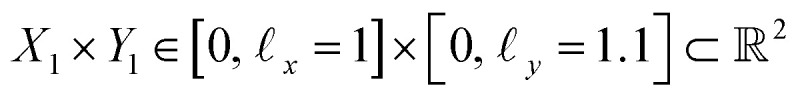
, where the operator × denotes the Cartesian product.^29^ For ease of illustration, we avoid degeneracy by ensuring that 
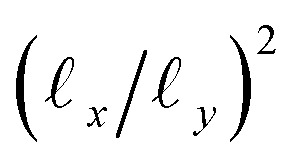
 is not a simple ratio.^28^ Again, we follow the DM method by calculating the pairwise distances between these points and embedding the data in a low-dimensional space. As demonstrated in Fig. 2C, the set of eigenvectors obtained in the smaller Gaussian bandwidth regime matched our expectations for the Laplacian eigenfunctions on a rectangular domain with Neumann boundary conditions. These canonical eigenfunctions are2
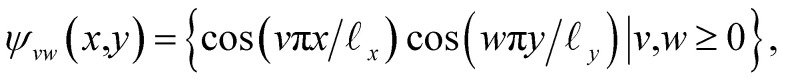
which follow the same pattern for higher-dimensional domains 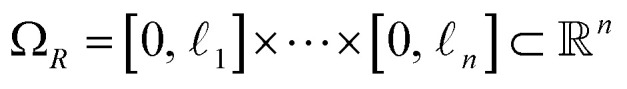 (with 
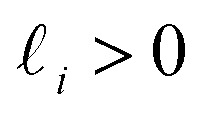
).^28^ Again, as we incrementally increased the Gaussian bandwidth from *ε*_↓_ to *ε*_↑_, this set of complete and orthogonal cosines smoothly transformed into the set of orthogonal Legendre polynomials, which are now functions of both *x* and *y*, as expected (Fig. 2D). Importantly, the leading Legendre polynomials provide a direct linear mapping of the input data points, which is a consequence of the linear terms *P*_1_(*x*) and *P*_1_(*y*). While these linear relationships are more convenient than the cosine form, we will show in Section 2.3 and 2.4 that they are absent in the embeddings of 3D EDMs and 2D projections.

It is important to take notice that, alongside leading eigenfunctions in the first two rows of each subplot in Fig. 2, the leading composites of these eigenfunctions {Ψ_*i*_ × Ψ_*j*_ | *i* < *j*} have also been plotted in the rows that remain, with each composite forming a unique 2D subspace. Mathematically, each such mapping to a 2D subspace is the restriction to the *N*-dimensional embedding of the projection of 
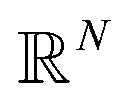
 onto 
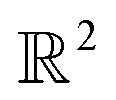
; given by {Ψ_1_ × Ψ_2_ × … × Ψ_*N*_} → {Ψ_*i*_ × Ψ_*j*_} (for expediency, we will use the term subspace to specifically refer to a subspace of an embedded manifold). Of interest, among the available subspaces, a leading parabolic trajectory exists for each degree of freedom present; for example, {Ψ_1_ × Ψ_4_} and {Ψ_2_ × Ψ_7_} in Fig. 2C, which correspond to the sequence of states along *Y*_1_ and *X*_1_, respectively. While less significant for the scope of the current section, the study and use of these 2D subspaces will be crucial in the sections to come, especially when dealing with experimental data, which we describe below.

Specifically, as the points in an experimental dataset naturally arrive in unordered sequence, one would have to properly sort the dataset indices to recognize the sinusoids shown in Fig. 2A and C; here, for example, there would be 50! sequences to consider. In the application, even if the ground-truth ordering was obtained, then in the presence of duplicate states (which we anticipate in an experiment), each sinusoid would be irregularly stretched along the horizontal axis where those duplicate states occurred, forming an unwieldy distorted sinusoidal form. However, as the points in each eigenvector are always scrambled in the same order for all eigenvectors, the composite of any two will always appear in a readily identifiable form. For example, as seen in Fig. 2A, a subset of the canonical Lissajous curves^31^ emerges across the 2D subspaces of each Ω_PD_, with the curves in this set having the form

3 *L*_*p*,*q*_ = {cos(*p*π*x*) × cos(*q*π*x*) | 0 ≤ *x* ≤ 1}

such that 
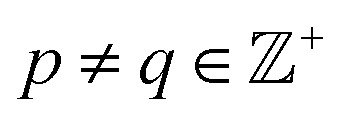
.

For these composites, we found that information pertaining to the given degree of freedom is portrayed most simply (without overlap) along a specific subset of *L*, here as seen across the set of 2D subspaces defined in pairwise combination with the leading eigenvector; *e.g.*, {(Ψ_1_ × Ψ_2_), (Ψ_1_ × Ψ_3_), …, (Ψ_1_ × Ψ_*z*_)}, where *z* is the index of the smallest non-zero eigenvalue. Specifically, this subset *T*_*k*_ ∈ *L* corresponds to the known Chebyshev polynomials of the first kind,^30^ of which we observed that the parabolic form is the lowest-order member present in each Ω_PD_ embedding.

**Fig. 3:**
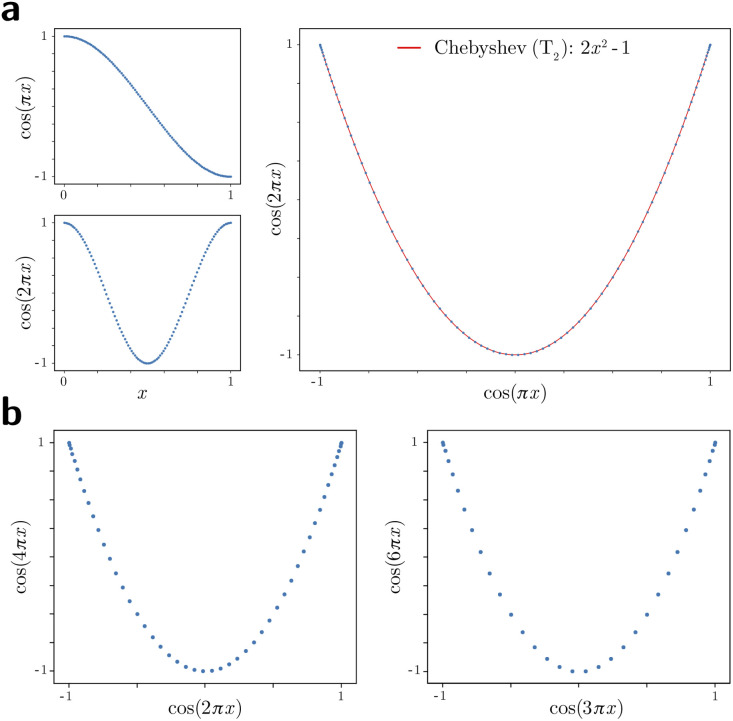
Analytical generation and analysis of Lissajous curves. The analytical generation of the Lissajous curve *L*_1,2_ = {cos(π*x*) × cos(2π*x*) | uniform *x* ∈ [0, 1]} is shown in (a). Note the naturally-induced nonuniform spacing between points near the boundaries and vertex of the parabola. As a simple demonstration, we also fit this curve with the Chebyshev *T*_2_ polynomial, which is a subset of the Lissajous curves; however, *T*_2_ does not share the same nonuniformity in spacing as *L*_1,2_. In (b), parabolic harmonics are likewise generated for *L*_2,4_ and *L*_3,6_. While the same *x*-coordinates were used to generate all underlying cosines for parabolas in both (a) and (b), more than one point in the domain ends up mapping to each coordinate of these parabolic harmonics. As such, these harmonics obfuscate the true conformational information, which is intact on *L*_1,2_.

Given their significance, these 2D subspaces have several important properties worth highlighting for their eventual use (or avoidance) during interpretation. First, note that for each sinusoidal subplot in Fig. 2A, points are equispaced along the horizontal axis in correspondence with the equidistance between points in the corresponding latent space. However, as a result of taking the Cartesian product of sinusoidal eigenfunctions, only non-uniform spatial relationships exist between neighbouring states in each *L*_*p*,*q*_; a relationship described by a non-isometric mapping^29^ where distances in a domain are not preserved in its codomain. As shown in Fig. 3, the spacing between points in *L*_1,2_, which is the composite of two such sinusoids, has an intrinsically nonuniform distribution. The density of points is similarly arranged as seen in the corresponding point clouds. For general reference, we denote this aspect with the term nonuniform rates of change.

Next for consideration, as seen for example in Fig. 2A, there exist several parabolic trajectories scattered throughout the 2D subspaces. However, only the first of these parabolas (here, {Ψ_1_ × Ψ_2_}) describes the full extent of the variational information present monotonically, while all other trailing parabolas (such as in the {Ψ_2_ × Ψ_4_} subspace shown) display a non-monotonic signal. As an analytical demonstration, Fig. 3 shows that the first three such parabolas can be generated *via L*_1,2_, *L*_2,4_ and *L*_3,6_. The latter two repeat the conformational information twice and three times, respectively, within one span of the parabolic trajectory.

As a consequence, only the mapping from the sinusoids to the first parabola in this set is bijective (injective and surjective),^29^ while all other mappings to higher-order parabolas are non-injective surjections. Importantly, since the Cartesian product of continuous functions is continuous, and so are projections from product spaces, this bijection further meets the requirements of a homeomorphism: a bijective correspondence that preserves the topological structures involved.^29^ We denote the higher-order parabolas (formed *via* the non-injective surjections) as parabolic harmonics, which do not preserve topological structure and must be avoided when determining a given degree of freedom; a problem that becomes more challenging as more degrees of freedom are added to the system.

In general, the presence of these patterns extends into latent *n*-spaces with *n* > 1. For every degree of freedom present in a state space SS_*n*_, there exists a corresponding set of Lissajous curves interspersed across specific {Ψ_*i*_ × Ψ_*j*_} projections of the corresponding embedding. Specifically, in the case of Fig. 2C, independently projecting the data for SS_2_ onto the planes spanned by its {Ψ_1_ × Ψ_*i*_} and {Ψ_2_ × Ψ_*j*_} combinations (where *i* > 1; *j* > 2) reveal a unique set of Chebyshev polynomials, with the sequence of points along these trajectories corresponding to CM_1_ and CM_2_. For convenience, a set of Chebyshev polynomials^30^ corresponding to a given CM will be referred to as its conformational modes). Thus, even though the knowledge required to view these sinusoids is unavailable outside of ground-truth studies, one can rely on their existence—*via* the composites of carefully chosen eigenvectors—to elucidate conformational type and order.

**Fig. 4:**
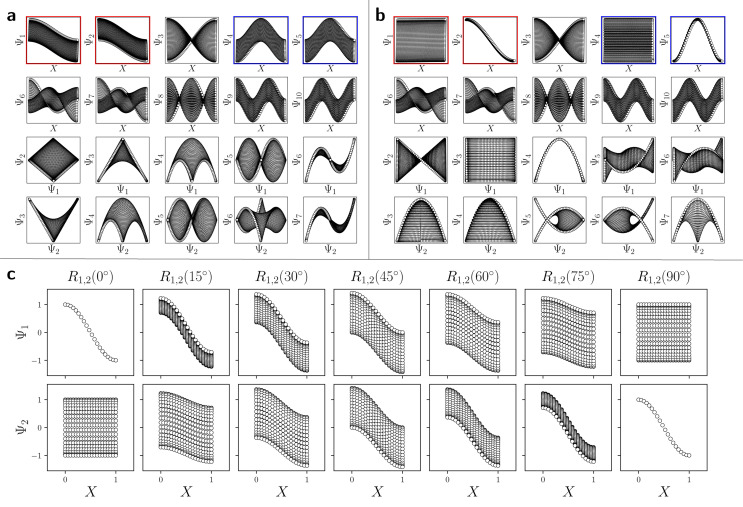
DM eigenvectors of the degenerate latent 2-space. DM eigenvectors of the *N* = 50 × 50 square domain for small Gaussian bandwidth (*ε*_↓_ = 5 × 10^−5^) are shown in (a) and (b) before and after high-dimensional rotations, respectively. It can be seen here that pairs of eigenfunctions exist that contain relationships aberrant to the canonical eigenfunction form seen in Fig. 2C. Two such pairs have been highlighted in red and blue, respectively, with the members of each pair always rotated 90° apart. To note, as any rotation can happen in the presence of degeneracy, this initial rotation is an arbitrary one. We demonstrate this property *via* the schematic in (c), which shows the angular relationship between two analytically-generated functions (cos(π*x*) and cos(π*y*), each displayed in the reference frame of states in *X*_1_) as they are jointly rotated 90°. By applying rotation operators *R*_1,2_(*θ*) = 45° and *R*_4,5_(*θ*) = 45° independently to two such aberrant pairs in (a), the canonical eigenfunction form begins to recover in (b), and more so as additional operators are appropriately applied.

With these details set aside, we next return to the smaller of the two Gaussian bandwidth regimes, in order to compare the previous nondegenerate rectangular results to those from a degenerate square domain, with *N* = 50 × 50 points equispaced identically along *X* and *Y* (Fig. 4A). Due to the presence of degenerate eigenvalues, which can arise for domains with a rational ratio 
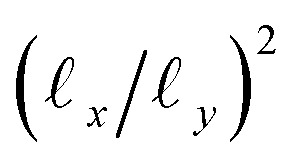
, we encounter pairs of eigenfunctions that appear different from the nondegenerate case of the rectangle.^28^ This can be seen, for example, by eigenvector pairs {Ψ_1_, Ψ_2_} and {Ψ_4_, Ψ_5_} in Fig. 4A. In Fig. 4C, we illustrate that these eigenfunctions are just rotated within their degenerate space, exactly as expected. We note that an eigenfunction associated with a degenerate eigenvalue is a linear combination of the degenerate eigenfunctions,^28^ where the normalization of the eigenfunctions restricts this linear transformation to a rotation and reflection (*i.e.*, the group of orthogonal transformations). For example, the {Ψ_1_, Ψ_2_} eigenvector pair is of form Ψ′ = *R*^T^Ψ such that4
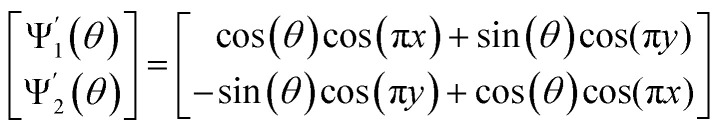


As seen for eigenvector Ψ_6_ in Fig. 4A, these summands can also have the form of two products: Ψ_6_ = *β*_1_ cos(π*x*) cos(2π*y*) + *β*_2_ cos(2π*x*) cos(π*y*), with any *β*_1_ and *β*_2_ such that *β*_1_^2^ + *β*_2_^2^ ≠ 0. Hence, it can be seen that these aberrant eigenfunction pairs are defined by an admixture of cosines in a higher-dimensional space, with form

5 Ψ_*i*_ = *β*_1_ cos(*v*π*x*) cos(*w*π*y*) + *β*_2_ cos(*w*π*x*) cos(*v*π*y*) = *β*_1_*ψ*_*vw*_ + *β*_2_*ψ*_*wv*_

By using an appropriate rotation operator *R*_*i*,*j*_, the summands within each eigenfunction pair can be maximally separated between two members Ψ_*i*_ = *ψ*_*vw*_ and Ψ_*j*_ = *ψ*_*wv*_, such that the canonical eigenbasis is recovered (Fig. 4B). As demonstrated using analytical expressions *ψ*_1,0_ = cos(π*x*) and *ψ*_0,1_ = cos(π*y*) in Fig. 4C, this separation occurs multiples of *θ* = 90° apart. In the Fig. 4C example, at *R*_1,2_(45°), these eigenfunctions have the form6
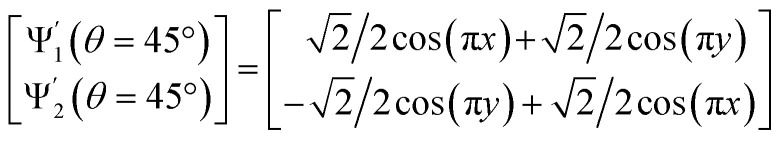
which decouples back into two distinct modes (*i.e.*, cos(π*y*) and cos(π*x*) for Ψ_1_ and Ψ_2_, respectively) at *R*_1,2_(90°). A similar result is obtained by applying this operation on the appropriate eigenvectors obtained *via* DM, with each initially assuming a random rotation angle (Fig. 4A) requiring a specific correction *R*_*i*,*j*_(*θ*), as seen in Fig. 4B.

While degeneracy is a coincidence which can be directly identified from the eigenvalue spectrum, a similarly rotated appearance (*i.e.*, eigenfunction misalignment) will later turn up during our investigation of PD manifolds. Pairs of misaligned eigenfunctions, at least approximately, can also appear when domains have undergone certain elementary geometric transformations. For example, by performing an affine transformation on a rectangle Ω_R_ to form a parallelogram Ω_P_, we observed a rotation of the first two eigenvectors, as similarly seen in Fig. 4A. Recall that an affine mapping preserves collinearity and ratios of distances, but in general not distances and angles.^32^ In Section 2.3, we will explore the possibility of other classes of transformations.

**Fig. 5:**
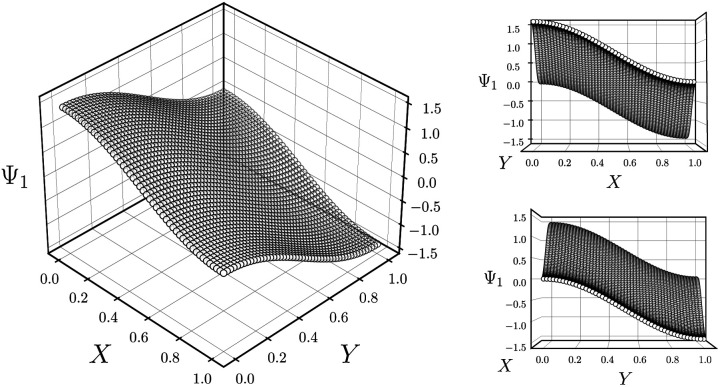
Intuition for sequential ordering of eigenfunctions based on ground truth. Subplots are analytically generated so as to match the appearance of Ψ_1_ in Fig. 4A. For this presentation, the equation Ψ_1_ = cos(*θ*) cos(π*x*) + sin(*θ*) cos(π*y*) was used with *θ* = 45°. As seen in the largest subplot, the eigenfunction exists in an *n*-dimensional space defined by the *n* degrees of freedom of the system. By displaying points in sequence corresponding to a known degree of freedom, here *X* or Y, we are effectively viewing each eigenfunction on a projected plane in its *n*-dimensional space.

As a final point in this section, we illustrate our method for retrieving the canonical eigenfunctions buried within an embedding, which has been used in Fig. 2 and 4, and extensively throughout the remainder of this work. Fig. 5 provides a schematic using the known analytical eigenfunctions chosen so as to match the results from DM on the square (degenerate) Ω_R_. As shown, a given sequence corresponding to the ground-truth arrangement of points along each degree of freedom (here *X*_1_ or *Y*_1_) captures the eigenfunction on a projected plane in the *n*-dimensional space where it resides.

### 2.2. Eigenfunctions of the 3D atomic-coordinate structures

We next investigate the manifolds obtained from the state spaces formed from a quasi-continuum of atomic-coordinate structures, each represented by a set of *m* 3D atomic-coordinates (*e.g.*, as visualized in Fig. 1). Importantly, the set of these 3D atomic-coordinate structures in 
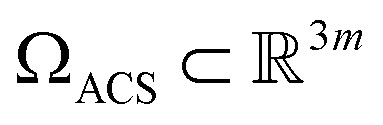
 represents the structural identity of each state, from which the cryo-EM experiment could only obtain two-dimensional information in the form of images.

#### 2.2.1 State space 2

Following the DM approach, we first calculated the distance matrix for SS_2_, which we obtained by the root-mean-square deviation (RMSD) for each pair of the *M*_2_ = 400 atomic-coordinates structures (as given by their PDB files). The RMSD between two atomic-coordinate structures *A* = (*a*_1_, *a*_2_, …, *a*_*m*_) and *B* = (*b*_1_, *b*_2_, …, *b*_*m*_), each composed of *m* atoms, is defined as7
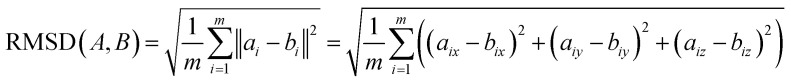
which is up to a trivial constant factor of *m*^−1/2^ equal to the Euclidean distance eqn (1).

**Fig. 6:**
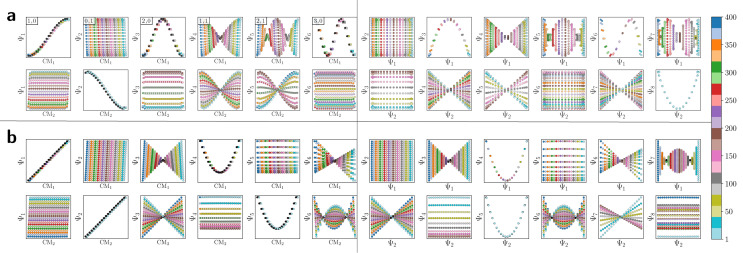
DM eigenvectors for the quasi-continuum of atomic-coordinate structures. Eigenvectors obtained for 20 × 20 = 400 atomic models occupying SS_2_ for small (*ε*_↓_ = 0.1) and large (*ε*_↑_ = 1000) Gaussian bandwidths are shown in (a) and (b), respectively. Leading eigenvectors are displayed in the first and second rows *via* sequential indexing along the ground-truth CM_1_ ∈ [1, 400] coordinates (*i.e.*, equispaced rotations of chain A) and CM_2_ ∈ [1, 400] coordinates, respectively. The color mapping illustrates the position of ground-truth states, where CM_1_ points can be seen following along the full spectrum of colors (indices 1–400) while CM_2_ points are approximately uniform in color map value (*i.e.*, light blue for points in front, with all other colors similarly underlaid with indices a multiple of 1–20). In (a), the modes {*v*,*w*} corresponding to each eigenvector are provided in the top left-hand corner, showing exceptional agreement with the LBO eigenfunctions on a rectangular domain with Neumann boundary conditions. We additionally note the absence of any significant eigenfunction misalignments.

The resulting DM embeddings for the small and large Gaussian bandwidth regimes, which are shown in Fig. 6A and 6B, respectively, share a strong resemblance with those found for the latent space (Fig. 2). Again, we note the presence of cosine eigenfunctions for the small Gaussian bandwidth regime, and a nearly-perfect linear form (*via* leading Legendre polynomials) in the large Gaussian bandwidth regime; *i.e.*, {Ψ_1_, Ψ_2_} in Fig. 6B. For the latter, we will show that such a linear form cannot be obtained in the other data types, 3D EDMs and 2D PDs, to be explored. In the small Gaussian bandwidth regime, we can identify both CM_1_ and CM_2_ parabolas residing in the subspaces {Ψ_1_ × Ψ_3_} and {Ψ_2_ × Ψ_8_}, respectively. Similar results—albeit for different dimensions—were found for the Ω_ACS_ embeddings from SS_1_ and SS_3_.

Briefly, we note that the results of PCA on this same dataset most closely resembles the geometries observed for DM in the large Gaussian bandwidth regime (in this instance, Legendre-like), with a slightly less uniform distribution. This suggests that DM better approximates intrinsic relationships in the data, as expected. A general similarity between the geometries observed using PCA and DM in the *ε*_↑_ regime will continue to manifest throughout the remaining sections of this article.

The striking similarity between the eigenfunctions of the latent space and the eigenfunctions of the atomic models can be rationalized as follows. Provided that the range of a single-body rotation is moderate (≲30°), the distance *D*_*ij*_ between any two states *i* and *j* within this range is to a good approximation 
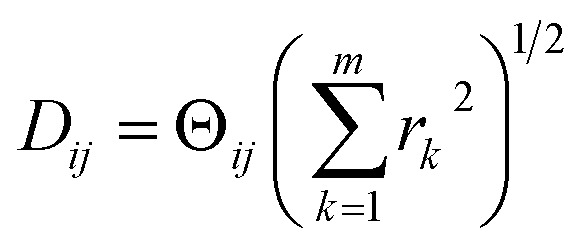
, where *r*_*k*_ is the distance of atom *k* away from the rotation axis, *m* the number of atoms of the body, and 
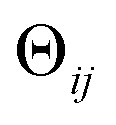
 the angular difference between the states. Therefore, *D*_*ij*_ is to a good approximation directly proportional to 
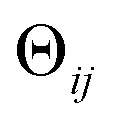
. If there are multiple independent body rotations (*i.e.*, CMs) present, the individual distances add in quadrature as in a Euclidean space. While not further discussed in this article, the linearity also holds for rigid body translations, where the distance is precisely proportional to the magnitude of the translation. Thus, the agreement between the eigenfunctions of the latent space and the ones of the atomic models is a direct consequence of the approximately linear relationship between distance and extents of multi-body motions (*i.e.*, moderate rotations and translations of any magnitude).

### 2.3. Eigenfunctions of the 3D density maps

We next investigated how the conformational relationships between states are changed when representation of structures by atomic coordinates is transformed into one by 3D electron density maps (EDMs; as visualized in Fig. 1). To this end, we generated the EDMs for each of the 3D atomic-coordinate structures for all previously-defined state spaces. We next calculated the pairwise Euclidean distances between these EDMs in 
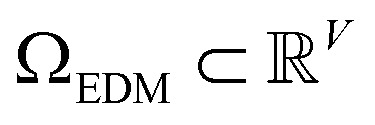
, with *V* the number of voxels, and performed an embedding *via* the DM method.

#### 2.3.1 State space 2

Over a wide range of Gaussian bandwidths, the structure of the resulting eigenfunctions is very similar to the structure of eigenfunctions retrieved for the atomic models in the small Gaussian bandwidth regime. Importantly, as in Section 2.2, these eigenfunctions are still of the form *ψ*_*vw*_, with subspaces having no significant appearance of eigenfunction misalignments.

However, there are a few attributes to consider that distinguish the manifolds obtained for EDMs from those retrieved for the previous data types. First, the difference between small and large Gaussian bandwidth regimes is much less drastic, such that only the cosine eigenfunctions appeared in both regimes. For small Gaussian bandwidth regimes (*i.e.*, a few orders of magnitude below the optimal value 
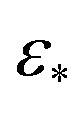
 determined by the bandwidth estimation method), we found that the leading CM_2_ eigenfunctions are buried deeply in low-ranking eigenvectors (*e.g.*, Ψ_8_ and higher), with numerous CM_1_ eigenfunctions occupying the eigenvectors in between. In addition, eigenvectors with cross terms Ψ_*i*_ = {*ψ*_*vw*_ | *v*,*w* ≠ 0} were scattered mostly in mid-range positions (*e.g.*, Ψ_12_ and higher).

In contrast, for larger Gaussian bandwidth regimes (*i.e.*, near and significantly above 
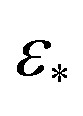
), eigenvectors with cross-terms are buried in much deeper subspaces (*e.g.*, Ψ_34_ and deeper), with the majority of leading eigenvectors housing content exclusively for either CM_1_ (*w* = 0) or CM_2_ (*v* = 0). These CM eigenfunctions also have a near-perfect distribution of points, whereas for the *ε*_↓_ regime, the distribution of points has noticeably less precision to the ideal form. Notably, the embeddings obtained above and below these regimes are incoherent in form.

**Fig. 7:**
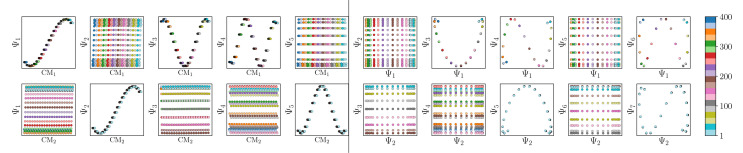
DM eigenvectors for the quasi-continuum of electron density maps. The results of the DM embedding of pristine EDMs from SS_2_ are shown. Leading eigenvectors as indexed by CM_1_ ∈ [1, 400] and CM_2_ ∈ [1, 400] are displayed in the first two rows on the left, followed by their composites on the right. Overall, there is near-perfect alignment of these eigenvectors with the canonical eigenfunctions, such that no eigenvector rotations are required. As an aside, the pronounced inward curling at the boundaries of certain subspaces (*e.g.*, {Ψ_1_, Ψ_3_}) is due to insufficient sampling.

We conclude that the eigenfunctions obtained from the larger Gaussian bandwidth regime would be preferred for several reasons. First, the desired CM_1_ and CM_2_ parabolas occupy leading subspaces and are thus easily identifiable. The paucity of leading cross-term eigenfunctions is also convenient, since they provide no useful information for our analysis while also obfuscating our search for desired subspaces. Additionally, the geometric structure of all subspaces obtained *via ε*_↑_ consistently appears much closer to the canonical form. In Fig. 7, we display the DM eigenfunctions obtained from this regime for the 20 × 20 = 400 EDMs occupying SS_2_. Subspaces indexed in the CM_1_ reference frame (row one on the left in Fig. 7) and the CM_2_ reference frame (row two, left) are displayed, as well as a set of leading composites of these eigenfunctions—forming 2D subspaces—on the right-hand side of the figure.

Importantly, as there was no Gaussian bandwidth value that could ‘recover’ the preferred Legendre-like form, it appears that this feature is lost upon transformation from atomic models to EDMs, caused by a change in the metric (to note, the curved geometry formed by cosines was also in close agreement with the results of applying PCA on this same dataset). As a main agent for this distinction, the distance measure pertaining to EDMs is fundamentally different from the one for the 3D atomic coordinates. Instead of the 3D coordinate points that stand for the positions of atoms in each structure, the data for each EDM is represented by a 3D array of values, one at each voxel (Fig. 1). A key difference, then, is that in the latter case the displacement of atoms is no longer accounted for individually. Instead, every voxel in the 3D map of one state is now compared to a voxel at the same location in another state, with only changes in the value at each voxel entering the distance measure.

**Fig. 8:**
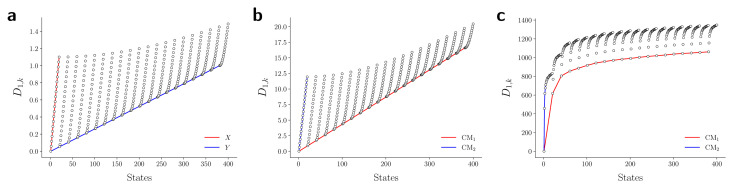
Comparison of metric for the three data types. The first row of the distance matrix ***D*** is plotted for the rectangular Euclidean space (a), the 3D atomic-coordinate structures in SS_2_ (b), and the EDMs in SS_2_ (c). Given our ordering of states, the first row *D*_1,*k*_ corresponds to the pairwise distance calculated between state 01_01 and all 400 states. For the pattern in (a), which was calculated on a rectangular domain Ω_R_ ∈ [0, 1] × [0, 1.1], one can identify the distance of the first state (*x*_1_ = 0, *y*_1_ = 0) to all other coordinates, such that the red line depicts the left-hand side of the rectangle (with maximum distance *D*{(*x*_1_, *y*_1_), (*x*_20_ = 1.1, *y*_20_ = 0)} = 1.1), and the blue line depicts the rectangle's base (with maximum distance 1). In (b), a similar rectangular pattern arises for the RMSD values calculated between atomic models. The pattern in (c), however, is starkly different from (a) and (b), such that no rectangular (or rectangle-like) domain could be drawn to reproduce this trend.

Hence, while the eigenfunctions are similar, the relationship between states in these two data types is fundamentally different. To demonstrate this change, Fig. 8 shows a comparison of the pairwise distances between states as calculated for the rectangular latent space, atomic-coordinate structures, and EDMs. As noted in the caption, by assessment of the close similarity between the distances from the latent space and atomic models, we can infer that these two data types are both confined to the rectangular manifold Ω_R_ (albeit of different sizes). More precisely, the metric space corresponding to ACS conformers in SS_*n*_ and to elements in *X*_*n*_ are very similar even though the two datatypes are very different. Since only the metric enters the LBO, their eigenfunctions are nearly identical.

In contrast, we see that the distances from the EDMs are starkly different from the rectangular pattern, where neighboring states are spatially arranged *via* an asymptotic-like trend. From these findings, we must infer that the corresponding data ‘live’ in an altogether different manifold. Although the explicit geometric form of Ω_EDM_ is unknown, we have shown that the leading eigenfunctions of the Laplacian on Ω_EDM_ are essentially preserved *via* the mapping from the latent space. While detailed knowledge of Ω_EDM_ is certainly of interest, it is inconsequential here since our analysis only requires an understanding of the leading eigenfunctions of a manifold.

### 2.4. Eigenfunctions of the 2D projections

Before providing a detailed description of the eigenfunctions of the LBO on 
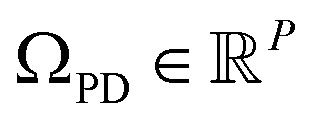
, with *P* being the number of pixels, we first provide a general analysis of their relationship with those from previously-established datatypes (*i.e.*, latent *n*-space, ACS and EDMs). For similarities, as was observed for the EDMs, we found that eigenfunction characteristics could be broadly grouped into two classes *via* either the small or large Gaussian bandwidth regime. In either regime, the eigenfunctions of the PD manifolds are again of the form *ψ_vw_*, such that only cosines emerge. The lack of the Legendre-like form and a similar asymptote-like appearance of distances between images suggests that the PD states in Ω_PD_ reside on a manifold similar to Ω_EDM_.

The overall difference between eigenfunctions obtained *via ε*_↓_ and *ε*_↑_ is also much more relevant for PDs than for the EDMs. In the small Gaussian bandwidth regime, CM_2_ subspaces exhibit a severely suboptimal point distribution, such that in some PDs, identification of the CM_2_ parabola-housing 2D subspace is completely obstructed. These CM_2_ subspaces are also buried in trailing eigenvectors and interspersed among those with cross-terms. We also note that the value determined by the bandwidth estimation method (
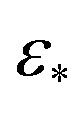
) provides similar spectral properties, making it a suboptimal choice for pristine data. In contrast, the large Gaussian bandwidth regime (*i.e.*, one order of magnitude larger than 
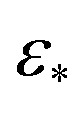
 and spanning numerous orders of magnitude above it) proves superior in every sense, with CM_1_ and CM_2_ eigenfunctions having ideal point distributions and corresponding subspaces occupying leading eigenvectors. And again, the cross-term eigenfunctions are present only in far trailing eigenvectors (*e.g.*, Ψ_31_ and higher), and would thus not be obstructive during the analysis.**Upon introduction of noise (SNR of 0.1) and *τ* = 5 noisy duplicates of each state, 
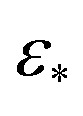
becomes the most suitable choice (along with numerous orders of magnitude above it), with anything below this range completely inadequate.

**Fig. 9:**
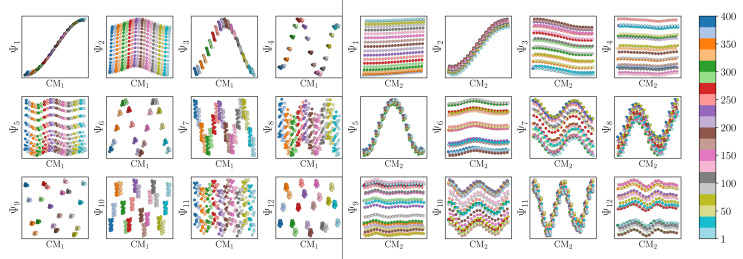
Analysis of eigenfunctions for an example PD in SS2. On the left are the sinusoidal forms cos(*k*π*x*) that emerge for only a specific subset of eigenvectors {*k* = 1, 3, 4, 6, 7, 9, …} when points in each Ψ_*k*_ are ordered precisely in the sequence of CM_1_ ∈ [1, 400] as assigned when the ground-truth images were initially constructed. Likewise, on the right, when points in each Ψ_*k*_ are instead ordered in the sequence of CM_2_ ∈ [1, 400], a new set of sinusoids emerge {*k*′ = 2, 5, 8, …} specifically for those remaining Ψ_*k*_ not in the previous CM_1_ subset. Hence, it can be seen that by systematically ordering the points in each eigenvector in sequence along each degree of freedom present, the corresponding set of sinusoids emerge in the frame of reference of the given degree of freedom.

Regardless, for either Gaussian bandwidth regime, we found that significant eigenfunction misalignments can emerge—with varying magnitude—depending on the direction of projection. The eigenfunctions of an example PD, determined with an appropriate choice of Gaussian bandwidth, are shown in Fig. 9. By plotting the points in each eigenvector in the specific ground-truth sequence constructed for CM_1_ against a uniform index (for the *M*_2_ states in SS_2_ indexed *via* {1, 2, 3, …, 400}), a similar display of sinusoids and grid-like patterns emerges as seen in Fig. 7, albeit now misaligned with the eigenvector basis (to note, PCA returned similar spectral features for all PD datasets explored, as demonstrated extensively in our companion article).^22^

Since we have previously shown that no such property is apparent in the manifold embeddings generated from the 3D EDMs from which these PDs originate, this suggests that the emergence of these eigenfunction misalignments must be tied to a changed metric in the PD manifolds. Specifically, as different 2D projections are taken of the EDMs *via p*: Ω_EDM_ → Ω_PD_, the geometry of Ω_EDM_ can become contorted due to the change of pairwise interatomic distances resulting from foreshortening in projection (Fig. 13 in ESI†), such that the apparent span of one CM to another depends on PD. We next describe these abnormalities in greater detail, before quantifying them exactly.

First, it is unclear what effect these eigenfunction misalignments may have on interpretability, since the frames of reference used so far (*e.g.*, as demonstrated in Fig. 9) are unavailable without *a priori* knowledge. To understand the effect of these eigenfunction misalignments on the PD manifolds, a collection of Ω_PD_ embeddings are next analyzed in greater detail and introduced in sequence of increasing intrinsic dimensionality. Notably, the explicit expressions derived in the previous sections will be used to account for the geometric structures observed in each PD embedding, which generally describe perturbations of a hypersurface spanned by the quasi-continuous conformations.

For the following analysis, sets of pristine images in five chosen PDs are obtained. The first PD is chosen to be normal to the plane of the CM_1_ rotation, such that all CM_1_ motions from that perspective only produce changes in the plane of the projection. A similar choice is made for PD_2_, which is projected onto the plane of CM_2_ motions (CM_2_ only exercised for *n* > 1), with the remaining three PDs chosen along arbitrary directions. For each state space, a set of these *M_n_* images is generated from one of these five PDs to form an *n*-manifold Ω_PD_ existing in a high-dimensional space 
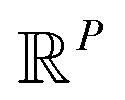
 (as a specific example, for SS_1_, there are five PD manifolds with 20 images each, while for SS_2_, a separate set of five PD manifolds are formed with 400 images each). We next embed data *via* the DM method with Euclidean metric and, using the eigenfunctions of the LBO, analytically quantify the trajectories of our simulated conformational changes as embodied by the spectral geometry of each Ω_PD_.

#### 2.4.1 State space 1

**Fig. 10:**
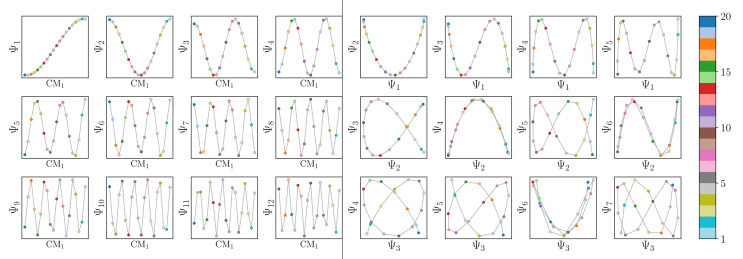
Analysis of eigenfunctions for PD_1_ in SS_1_. On the left are the sinusoidal forms *ψ_k_* that emerge when points—corresponding to images—in each eigenvector are ordered precisely in the sequence CM_1_ ∈ [1, 20] in which their ground-truth images were constructed. Regardless of any knowledge of such a sequence, the composites of these eigenvectors will always form well-defined geometries *via* the Lissajous curves, as shown on the right. In the first row on the right are the Chebyshev polynomials of the first kind, of which the parabola {Ψ_1_ × Ψ_2_} is the simplest mapping of the conformational information present. Figure source: Institute of Electrical and Electronics Engineers (IEEE) article^22^ (with cosmetic alterations) under licence CC BY 4.0.

We first generated a different DM embedding using a suitable *ε* value within the range discovered for each of the five PD manifolds in SS_1_. Each of the resultant point clouds contains 20 points, with each point corresponding to an image of a conformational state from CM_1_. We next ordered the eigenvector coordinates in each Ω_PD_ embedding to correspond to the ground-truth sequence of CM_1_ states; *i.e.*, as understood *via*Fig. 5. As anticipated given our previous analysis, we observed the canonical eigenfunctions of the LBO on the interval [0, 1] subject to Neumann boundary conditions8
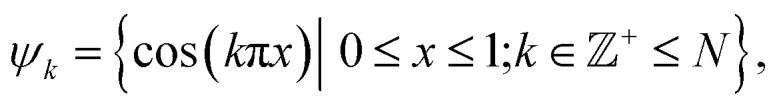
where *x* is the conformational coordinate represented by a number in the interval [0, 1]. For demonstration, we plot each of the 1D points in a given eigenvector as a function of a uniform index *I* ∈ [1, 20] (for the total of 20 states in SS_1_), making sure that the ordering of points in 1D follows the sequence assigned by the ground-truth index of its corresponding image along CM_1_. As seen in Fig. 10, when the collection of points in each eigenvector are ordered appropriately, the eigenfunction's sinusoidal form emerges along the full extent of the degree of freedom present (*i.e.*, mapping *I* ↦ *x* ∈ [0, 1]).

We next compared these sets of 2D subspaces among the five PDs, and found only subtle differences in the distribution of their point clouds. It is important to underscore here the natural discrepancies between different PD manifolds that should be expected—due to what we term PD disparity—which will continue to manifest in several significant forms throughout this analysis. Naturally, as each 2D projection provides an incomplete representation of the underlying 3D density map, depending on the type of motion and its component on the PD under investigation, ground truth is preserved to different degrees. This disparity affects all Ω_PD_ characteristics and will become more relevant as we investigate the embeddings of datasets generated from structures with multiple degrees of freedom.

#### 2.4.2 State space 2

To further understand these trends for embeddings formed with increasing intrinsic dimensionality, we next investigated the embeddings generated for SS_2_. As previously shown in Fig. 9A, by plotting the points in each eigenvector in the specific ground-truth sequence constructed for CM_1_ against a uniform index, a similar but now interspersed pattern of sinusoids appears. Specifically, the appearance of the sinusoids (with increasing 
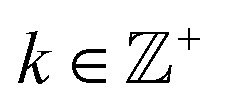
) only manifest in a subset of all eigenvectors present, while for all other eigenvectors grid-like patterns emerge. These findings concur with our analysis throughout Section 2, where it was shown that such patterns arise as a consequence of viewing each eigenfunction on a projected plane in *n*-dimensional space (Fig. 5).

As a demonstration of this property, we next reordered the indices of points within all eigenvectors to instead correspond to the specific ground-truth sequence constructed for CM_2_ (*i.e.*, {1, 21, 41, …, 381}, …, {20, 40, 60, …, 400}). The output of this operation can be seen in Fig. 9 on the right, which manifests a new subset of interspersed sinusoids, with increasing 
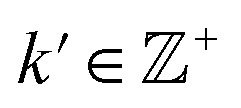
 independent from the previous subset; and inhabiting only those eigenvectors in the complement of the CM_1_ subset. By induction—based on these observations and those in Section 2.1—we conclude that for *n* degrees of freedom in a given Ω_PD_, there are *n* independent sets of sinusoids *ψ*_*k*_. Each set 
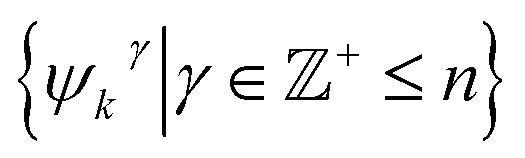
, denoted by an index *γ* per degree of freedom, is interspersed throughout the collection of available eigenvectors {Ψ_*i*_ | *i* ∈ *N*}.

**Fig. 11:**
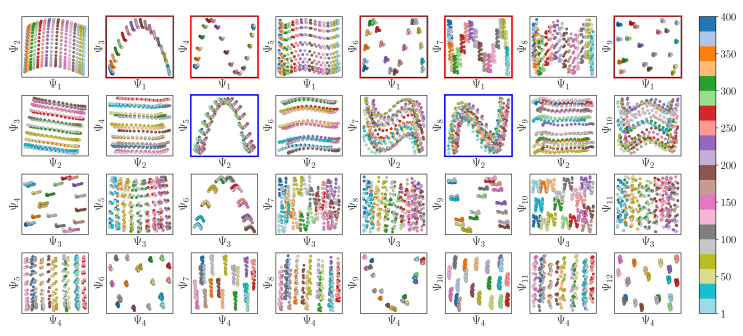
A subset of the space of 2D subspaces for PD1 in SS2. As demarcated in red and blue boxes, a set of conformational modes exists for both CM_1_ ∈ [1, 400] (red boxes, {Ψ_1_ × Ψ_*i*_}) and CM_2_ ∈ [1, 400] (blue boxes, {Ψ_2_ × Ψ_*j*_}); where *i* > 1 and *j* > 2), interspersed throughout each row. Additionally, note the occurrence of the first parabolic harmonic for CM_2_ located at {Ψ_3_ × Ψ_6_}. Similar plots for the remaining four PDs are provided in (Fig. 14 in ESI†). Figure source: Institute of Electrical and Electronics Engineers (IEEE) article^22^ (with cosmetic alterations) under licence CC BY 4.0.

Also, in agreement with Section 2.1, we found that for every CM present in the state space, there exists a corresponding set of Chebyshev polynomials. Specifically, in the case of PD_1_, independently projecting the data for SS_2_ onto the planes spanned by its {Ψ_1_ × Ψ_*i*_} and {Ψ_2_ × Ψ_*j*_} combinations (where *i* > 1; *j* > 2) revealed a unique set of these polynomials, with the sequence of points along these trajectories corresponding to CM_1_ and CM_2_ (Fig. 11). With this knowledge in hand, we next compared the subset of eigenfunctions as obtained in either the reference frame of CM_1_ or CM_2_ (Fig. 9, left or right-hand side, respectively) with the Chebyshev polynomials in Fig. 11.

Indeed, each of the Chebyshev polynomials mapping CM_1_ information in Fig. 11 (visualized with subplots enclosed by blue boxes) corresponds to the subset of sinusoidal eigenfunctions that emerged in the reference frame of CM_1_ in Fig. 9A; with similar relations holding for CM_2_ in Fig. 9B. Combining this empirically-obtained knowledge with our *a priori* understanding of the eigenfunctions of the LBO on known domains as understood throughout Section 2, we are able to express the analytical form of these Ω_PD_ eigenfunctions. In close approximation, we found that the leading Ω_PD_ eigenfunctions appear in the form

**Fig. 12:**
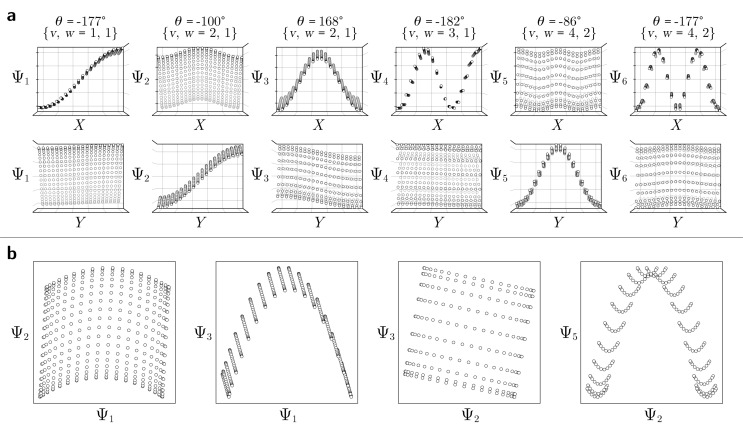
Comparison of analytically-generated functions with heuristic results previously obtained for PD_1_. For each pair of subplots, values for *θ* were approximated by eye. Our approximations in (a) and (b) share a remarkable similarity with heuristic results shown in Fig. 9 and 11, respectively, and are able to account for geometric minutiae previously unaccounted for, as well as larger-scale rotations seen in the composite of eigenfunctions. For example, discrepancies can be seen in the slightly tilted appearance of the geometry in Ψ_3_ of Fig. 9, and corresponding curling at the edges of {Ψ_2_, Ψ_3_} in Fig. 11. These differences can be understood as additional, small-scale perturbations which are currently unaccounted for in our general expression.

9 Ψ_*i*_ = cos(*θ*) cos(*v*π*x*) + sin(*θ*) cos(*w*π*y*) = *β*_1_*ψ*_*v*_ + *β*_2_*ψ*_*w*_,

such that a given eigenvector Ψ_*i*_ may contain some linear combination of the *n* canonical eigenfunctions 
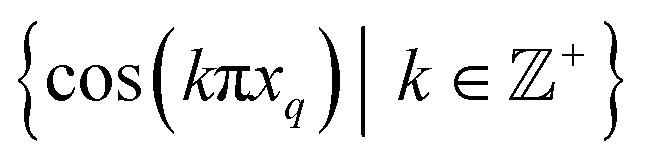
 corresponding to the *n* degrees of freedom 
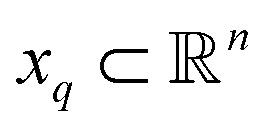
. As shown in Fig. 12, we were able to use this explicit expression to near-perfectly emulate the heuristic results obtained in Fig. 9 and 11.

As also demonstrated in our analysis of eigenfunction misalignments in Section 2.1, the sum of these squared coefficients is conserved across pairs of eigenvectors, such that the base functions Ψ′_*i*_ = *ψ_v_* and Ψ′_*j*_ = *ψ_w_* can be expressed as a rotation Ψ = *R*^T^ Ψ′, having the form10
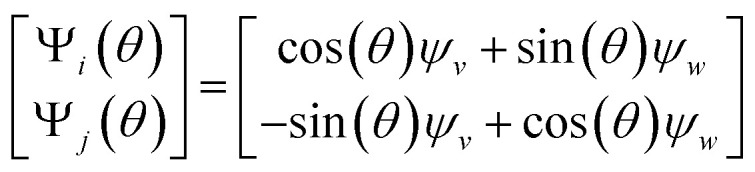


This analytical expression suggests that conformational information—pertaining to each of the system's degrees of freedom—will lie on some linear combination of the embedded manifold's orthogonal eigenvectors. This feature is seen most strikingly in {Ψ_3_ × Ψ_4_} of Fig. 14B,† where the parabolic surface described by the Chebyshev polynomial is significantly out of alignment with the plane of the 2D subspace containing it. Similar instances, albeit in more subtle form, also arise for surfaces in the remaining three PDs of Fig. 14† (to note, we have also included a brief analysis of state space 3 with similar findings; see Fig. 15 in ESI†). In Section 2, we demonstrated that the need for eigenfunction realignment is due to the change in apparent interatomic distances dependent on projection direction, as illustrated *via* Fig. 13 in ESI.† This disparity among PDs is inevitable and poses a fundamental challenge, which we have addressed using ESPER in our companion article.^22^

## 3 Analysis of complex physical constraints

Finally for consideration, we have so far only dealt here with molecular machines that specifically exhibit each of their domain motions along an independent and mutually unrestricted sequence of quasi-continuous states. All *n*-wise combinations of these bounded intervals (one for each conformational motion) produce an *n*-dimensional shape with a rectangular boundary. In our analysis, we have shown that the corresponding Laplacian eigenfunctions are well defined for this domain.

However, one can still imagine all sorts of other situations, such as a system where one domain blocks—*via* steric hindrance—another domain from its full range of motion in a specific region of the state space. Additionally to consider are state spaces with “holes” (*i.e.*, interior boundaries),^28^ where the occurrence of certain states is forbidden due to energetic restraints. Indeed, the presence of complex physical constraints can drastically change the boundary shape of the manifold's domain, and consequently its eigenfunctions. Less fundamentally restricted, similar aberrations can occur due to lack of coverage—as obtained by experimental measurements—of the state space. For example, obtaining poorly sampled data from a rectangular domain would allow any number of arbitrary shapes to emerge.

To better understand the effects of these boundary challenges within the scope of our heuristic analysis, we have created a 2D state space with an octagonal domain; noting that, in this case, analytical solutions of the Laplacian eigenfunctions are not available. The octagonal boundaries were created by eliminating states at the four corners of our standard rectangular domain, as shown in Fig. 16A in ESI.† To circumvent potential occurrences of eigenfunction misalignments due to PD disparity—which may complicate the interpretation of the boundary influence—we opted to embed the 3D electron density maps instead of 2D projections for this analysis. The corresponding manifold embedding obtained from this octagonal state space is shown in Fig. 16C,† which features a number of deviations from the canonical rectangular eigenbasis (Fig. 7). Manually, we attempted to find a transformation from the octagonally-derived eigenbasis (Fig. 16C†) to the rectangular form (Fig. 7) by assuming a collection of suitable rotation operators.††††We note that both the indices and number of rotation operators required for this transformation deviated from our PD-manifold findings on eigenfunction realignment performed on rectangular state spaces,^22^ creating a more complex tree of decisions. Indeed, we are able to show that such a transformation is possible, up to some level of uncertainty (Fig. 16D†).

## 4 Discussion

Through our analysis, we have shown how the embeddings of manifolds of experimental data originating from structures undergoing the same conformational changes vary depending on datatype. Specifically, we have identified how the spectral geometry transforms as the datatype is changed stepwise from 3D atomic-coordinate structures to 3D density maps to sets of their 2D projections. In our companion work,^22^ these findings for 2D projections have already been applied for developing an improved method, ESPER, to retrieve conformational variability of molecules and their free-energy landscape from ensembles of single-particle cryo-EM images. Similarly, this analysis could be used for the recovery of energy landscapes based on data from other measurement or simulation techniques, including ensembles of atomic models obtained by molecular dynamics (MD) simulations^33^ and 3D electron density|| maps obtained by cryo-electron tomography^34^ (cryo-ET).

During this exposition, we have laid out a framework for generating and analyzing ground-truth data across unique datatypes with detailed guidelines on how to extend our current scope to different experimental approaches. For example, these practices could be applied with minimal alterations to studies of conformational continuum tailored to single-particle imaging with an X-ray free-electron laser (XFEL),^35^ nuclear magnetic resonance spectroscopy (NMR),^36^ and time-resolved (TR) cryo-EM.^37^ Generally, we believe that there is a potential for the application of our methodology to a wide area of research, particularly where biological systems exercise multiple degrees of freedom in a continuous manner. Our companion article provides one such direct application of these findings to the study of single-particle cryo-EM data, where we describe a method for navigating the spectral geometry of 2D projection data in the presence of experimentally-relevant SNR and CTF to recover a quasi-continuum of 3D structures and corresponding free-energy landscape.^22^ Beyond the potential for additional applications, a comprehensive understanding of how the eigenfunction basis of a manifold changes dependent solely on datatype is an area of research with wide implications for machine learning across a diversity of fields.

More technically, we next summarize key assumptions of this framework that pertain to the relevancy and breadth of our heuristic analysis of structural heterogeneity. Of first consideration, we have focused here on situations where structures undergo collective rigid-body motions, which we believe are sufficient for the description of most molecular machines, but may fall short of addressing instances involving more complex situations. Examples are studies of machines entailing the concerted binding and release of ligands, which naturally require a separate state space for each possible combination of the machine with its binding partners. For such a situation, a similar heuristic analysis could be conducted using synthetic models combining two or more state spaces.

In our previous work,^22^ we further tested the ability of PCA and DM to correctly embed PD manifolds formed from models exercising more complex domain motions using the so-called “mouth-wing” toy model. Notably, the spheres making up the “mouth” domain in this toy model were uniquely positioned (keyframed) for each state so as to gradually clump together (ultimately presenting a higher density towards the fully-open state), while the “wings” were programmed to open in unison at constant angle increment about their hinge and curl inwards at the same time. Compared to the synthetic framework used to generate the Hsp90 dataset, the construction of mouth-wing data provides a radically different situation, and accounts for complex interactions of spheres within each domain motion, while still obeying conservation of mass. The embedding of the mouth-wing images still manifested all essential spectral characteristics detailed for Hsp90. When random positional noise was applied to each sphere across all mouth-wing models, changes to the spectral geometry were effectively no different than from applying additive Gaussian noise on the rendered image of each model. Although, with the addition of the mouth-wings model, we fall short of an exhaustive coverage of possible data models, we believe the correspondence between its outputs and those of the Hsp90 dataset created with entirely different design principles suggests the generality of our discoveries.

Finally, we will discuss the challenges of analyzing systems with complex physical constraints. For the majority of this study, we have been interested in casting a wide enough net so as to capture the dynamics of a large portion of conformer systems, which we surmise operate within rectangular boundaries of an *n*-dimensional latent space of relatively-rigid multi-body motions. However, as described in Section 4, one can still imagine other situations where the boundaries could admit any arbitrary shape. As an example, we demonstrated how the eigenfunctions change when our ground-truth data is constrained by octagonal boundary conditions (Fig. 16†). Furthermore, there exists a potential for complex relationships that render the manifold noncompact.^29^

In general, analytically solving the Laplacian for any arbitrary boundary is impossible, since analytical solutions are only available for certain elementary shapes such as rectangles, discs, ellipses and special triangles.^28,38^ On the other hand, spectral methods such as diffusion maps can efficiently obtain numerical solutions, in principle for any boundary. However, this could present additional challenges, since numerical solutions may be harder to incorporate in the process than analytical forms. Depending on the system under study, the prevalence of non-trivial boundaries inducing eigenfunction dissimilarity may vary, with a more detailed analysis required in each case.

Still, when studying systems described by a compact manifold, it is normally expected that the hypercube will continue to ‘fill in’ as more data are acquired from the experiment (or simulation in the case of MD). As previously addressed in Section 4, we contend that in the case of conformers of molecular machines, non-trivial boundaries are the exception rather than the rule. When available, it is most lucrative then for experimentalists to aspire towards acquiring this most complete information. In this way, they will maximize the ability to track conformational changes in their analysis.

## 5 Conclusions

The power of geometric machine learning stems from its potential to detect and follow the intrinsic geometry of a manifold of continuous conformations hidden in a large noisy dataset. In our application of geometric machine learning to experimental cryo-EM single-particle images of large ensembles of biomolecules, we have explored the feasibility of this approach by using simulated ground-truth data of a molecule exercising conformational motions with multiple degrees of freedom. Already, insights gained from our analysis have been used to substantially improve the ManifoldEM framework and its ability to retrieve continuous conformations and functional pathways from single-particle cryo-EM data. Beyond ManifoldEM, we have shown that our machine learning heuristics have the potential for application to data collected from other experimental methods, particularly from cryo-electron tomography (cryo-ET), X-ray free-electron lasers (XFEL), and molecular dynamics (MD) simulations. More generally, we demonstrate the importance of systematically analyzing simulated ground-truth datasets to inform on key features of different datatypes in geometric machine learning, which has significant implications for the reconstruction of complex information obtained from a wide range of experimental designs.

## Graph tools

All figures were made using some combination of Matplotlib,^43^ Adobe Creative Suite,^44^ Cinema4D,^45^ PyMOL,^46^ and ChimeraX.^47^

## Data availability

The Python data and code repository for reproducing this work is available on GitHub.^39^ As well, the software repository for the ESPER method is available on GitHub^40^ and on the IEEE DataPort.^41^ The IEEE repository is a mirror of the GitHub repository, while additionally containing a more extensive catalog of simulated data and results, including the atomic-coordinate structures and 3D density maps used to generate 2D projections of the Hsp90 continuum dataset. Synthetic datasets can likewise be generated from scratch using our synthetic data generation protocol.^42^

## Author contributions

Author contributions are listed alphabetically below. All authors reviewed the final manuscript. Conceptualization: ES, PS, JF. Formal analysis: ES. Investigation: ES. Methodology: ES, PS. Validation: ES, PS. Manuscript draft: ES. Manuscript review and editing: ES, PS, JF. Supervision and project administration: JF.

## Conflicts of interest

There are no conflicts to declare.

## Supplementary Material

DD-002-D2DD00128D-s001
