# Supplementary material for: Beyond ManifoldEM: geometric relationships between manifold embeddings of a continuum of 3D molecular structures and their 2D projections
Source: Digit Discov. 2023 Apr 6;2(3):702–17. doi: 10.1039/d2dd00128d (PMC10259371; doi:10.1039/d2dd00128d)
Supplement: DD-002-D2DD00128D-s001 [file DD-002-D2DD00128D-s001.pdf]

Electronic Supplementary Material (ESI) for Digital Discovery.
This journal is © The Royal Society of Chemistry 2023

Supplementary Material

A. Dimensionality reduction frameworks†

Diffusion Maps. First the pairwise distance between every pair of elements in the dataset is calculated to form a symmetric $N \times N$ distance matrix $\mathbf{D}$, where a single row represents the Euclidean distance of the corresponding row-indexed element to each column-indexed element. Using this matrix as input, the nonlinear-dimensionality reduction method diffusion maps[1] (DM) is next applied to generate the corresponding conformational manifold. To begin, an isotropic Gaussian kernel is applied to these distances to create a real, symmetric similarity matrix

$$A_{ij} = \exp(-D_{ij}^2 / 2\varepsilon) \tag{11}$$

The similarity matrix $\mathbf{A}$, calculated using a suitable ε value (i.e., the Gaussian bandwidth), is then divided by a diagonal matrix of its row sums to construct a symmetric, positive semidefinite stochastic Markov transition matrix $\mathbf{M}$. This matrix represents the relative pairwise affinity among all images and is closely related to the normalized graph Laplacian $\mathbf{L} = \mathbf{I} - \mathbf{M}$, where $\mathbf{I}$ is the identity matrix.[2] Eigendecomposition of the matrix $\mathbf{M}$ is then performed to retrieve an ordered set of N orthogonal eigenvectors ranked by eigenvalue, which define a nonlinear spectral embedding of the data.[3]

The Gaussian bandwidth in the above expression has a strong influence on the notion of similarity between images. An optimal Gaussian bandwidth value, henceforth denoted $\varepsilon_\star$, can be determined by a prominent routine—the "bandwidth estimation" method—which uses the correlation dimension as a measure of fractal dimensionality.[2–4] At small Gaussian bandwidths, the system takes on a relatively fine-grained definition of similarity (i.e., data points only see their direct neighbors). Increasing ε transforms this relationship into a more coarse-grained notion of similarity. These notions of similarity govern the behavior of all subsequent steps, and ultimately impact the geometric structure of the resultant manifold embedding.

Notably, in the limit $\varepsilon \to 0$ and $N \to \infty$, and with an appropriate normalization of the similarity matrix,[1] the DM eigenvectors converge to the eigenfunctions of the Laplace-Beltrami operator (LBO).[3] The LBO acting on a scalar function f on a Riemannian manifold is given by

$$\nabla^2 f = g^{-1/2} \partial_i (g^{1/2} g^{ij} \partial_j f) \tag{12}$$

where $g = \det(g^{ij})$ and g^{ij} are the components of the metric tensor.[5,6] Specifically, the eigenfunctions of the LBO, $\nabla^2 f = \lambda f$, form a complete basis in the function space $L_2(\Omega)$ of measurable and square-integrable functions on the manifold Ω.[7] For a bounded manifold, the eigenfunctions must further satisfy boundary conditions; for example, DM requires the Neumann boundary conditions,[1] such that the normal derivatives on the boundaries vanish. Therefore, the eigenfunctions depend also on the boundary of Ω.

It is well understood that the eigenfunctions of the LBO on Ω carry valuable information about the underlying intrinsic geometry and are thus important for understanding many systems. For compact manifolds with a boundary, as an example, the eigenfunctions are the modes of vibration of a 1D string or a 2D membrane. For compact manifolds without a boundary (i.e., closed manifolds), the well-known spherical harmonics are eigenfunctions of the spherical surface. In the field of structural biology, the eigenfunctions of the LBO on $\mathrm{SO}(3)$, which are the Wigner-D functions, have been used for retrieving the unknown orientations of single-particle X-ray and cryo-EM snapshots.[8,9] In general, the eigenfunctions of the LBO on different manifolds are fundamental to mathematics and sciences, and describe a wide diversity of seemingly disparate phenomena—reflecting the so-called "underlying unity of nature"—from quantum mechanics to gravitational fields.[10]

Principal Component Analysis. For the PCA approach,[11] instead of defining the Gaussian kernel as previously used in DM for the Markov transition matrix, a matrix $\mathbf{Z}$ of dimension $P \times N$ is formed, where P is the number of components (e.g., number of pixels when dealing with images) describing each element of the dataset. Additionally, $\mathbf{Z}$ is normalized by removing the mean of all images from each image. Finally, an eigendecomposition of the $N \times N$ matrix $\mathbf{Z}^T\mathbf{Z}$ yields a set of orthogonal eigenvectors, the principal components (PC), together with corresponding eigenvalues.

To note an important commonality between PCA and DM, the matrix $\mathbf{Z}^T\mathbf{Z}$ is symmetric and positive semi-definite (i.e., all eigenvalues are larger than zero),[12] which is also the case for the Markov transition matrix used in the DM method. A detailed comparison of results for PCA and DM for pristine PD datasets is provided in our companion article, where we further study the sensitivity of PCA and DM to experimental perturbations such as SNR and CTF.[13]

B. Additional properties of PD manifolds

Two orthographic views of 3D models in the directions of two PDs are shown in Figure 13-A and 13-B, each composed of 20 overlaid 3D volumes from CM_2. The 2D distances (in units of pixels) were measured between the peripheral ends of each consecutive states' rotated subunit (as seen in red and blue encircled regions). In Figure 13-C, the mean 2D distance measurements on each consecutive region (i.e., the red and blue region, respectively) are plotted with error bars representing standard deviation, along with linear regression. Although the interval between successive 3D states is constant, when projections are taken, apparent distances can strongly vary based on the viewing direction. We note that the behavior of the Euclidean distance matrix calculated in the DM method is less intuitive than the distance matrix in the current demonstration, and instead records the changes on a pixel-by-pixel basis for the entire image.

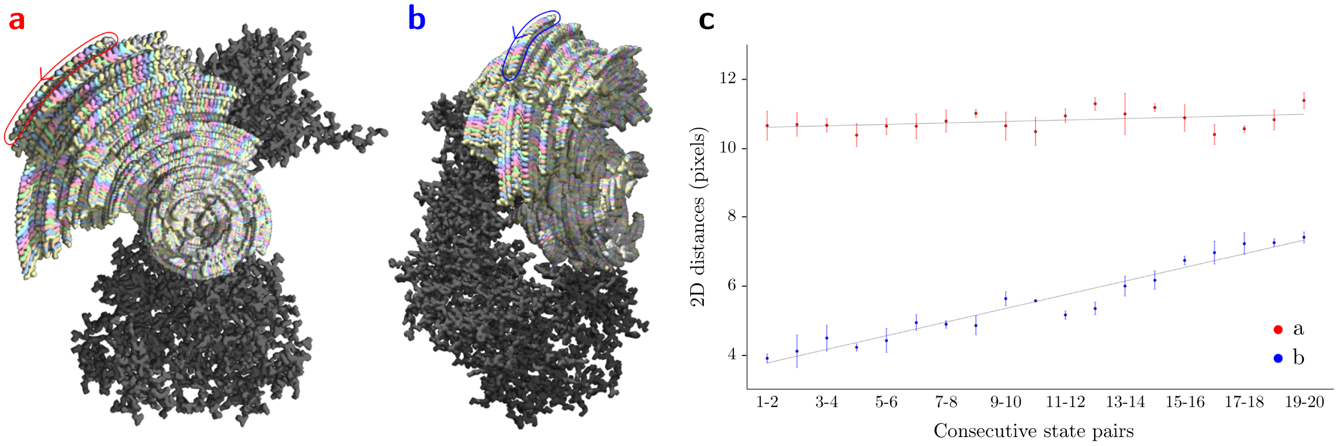

Fig. 13 Example for emergence of PD disparity due to foreshortened distances when taking 2D projections of 3D EDMs. To note, pixels were only tracked for demonstration purposes in the current figure, which is not a prerequisite for our unsupervised machine learning approach.

In Figure 14, a presentation similar to Figure 11 is shown for the remaining four PDs. Here, subspaces requiring eigenvector rotations (e.g., both parabolas in **b**) and presenting subtle boundary problems (e.g., the inwards curling of the point-cloud trajectory in $\{\Psi_3 \times \Psi_4\}$ of **d**) can also be seen in certain 2D subspaces. Note that for the PD in **a**, due to PD disparity, the hierarchy of CM information is actually reversed from those seen in the other four PDs. Here, the CM_2 Chebyshev polynomials are instead present along $\{\Psi_1 \times \Psi_i\}$ combinations (in the first row), while CM_1 Chebyshev polynomials are present along $\{\Psi_2 \times \Psi_j\}$ combinations (in the second row).

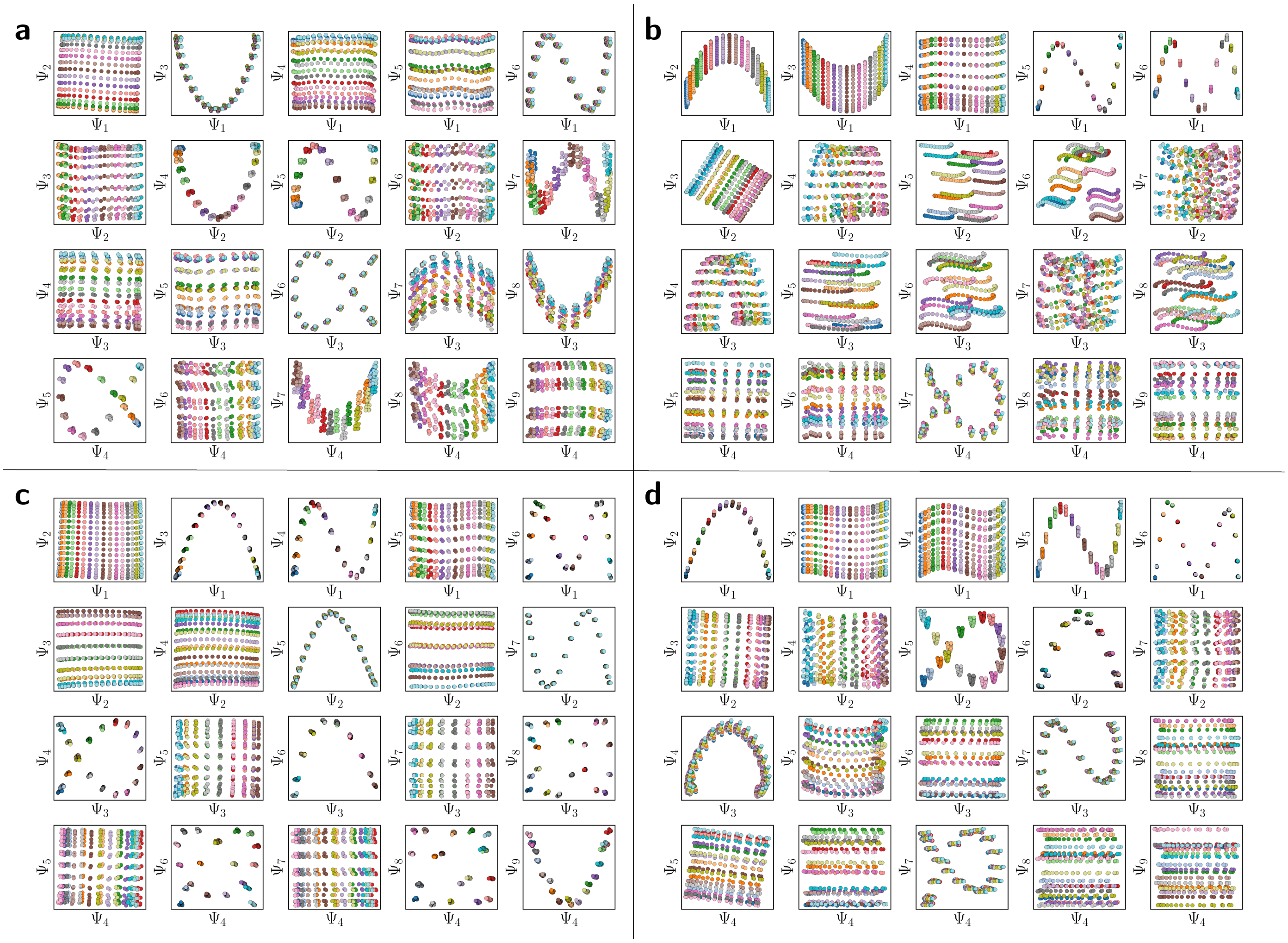

Fig. 14 Collection of 2D subspaces from leading eigenfunctions for the remaining four PDs, as was similarly presented for PD_1 in Figure 11.

We further investigated the $M_3 = 10 \times 10 \times 10 = 1000$ states making up SS_3 (chosen for ease of computation to contain only half as many states along each degree of freedom as compared to SS_1 and SS_2). For each conformational motion present in a given PD data set (this time for CM_1, CM_2 and CM_3), a set of unique Lissajous curves were again found spanning specific 2D subspaces of the embedded manifold, with the Chebyshev subset describing the corresponding CM along a trajectory in the 2D subspace explicitly. As an example, Figure 15 shows the set of 2D subspaces where these modes exist for PD_5. To note, due to the increased complexity of SS_3, these patterns were much more interspersed throughout the embedding, but still followed a similarly consistent ordering. In addition, due to the relatively small range of motion exhibited by the third conformational domain (as seen from these PDs and as designed in the ground-truth structures), all CM_3 modes were found in higher-order eigenvectors; e.g., Ψ_5 and higher for these five PDs. As similar patterns were identified in SS_3 as in previous accounts, for the remainder of our study, focus is honed onto mapping data sets generated for SS_2.

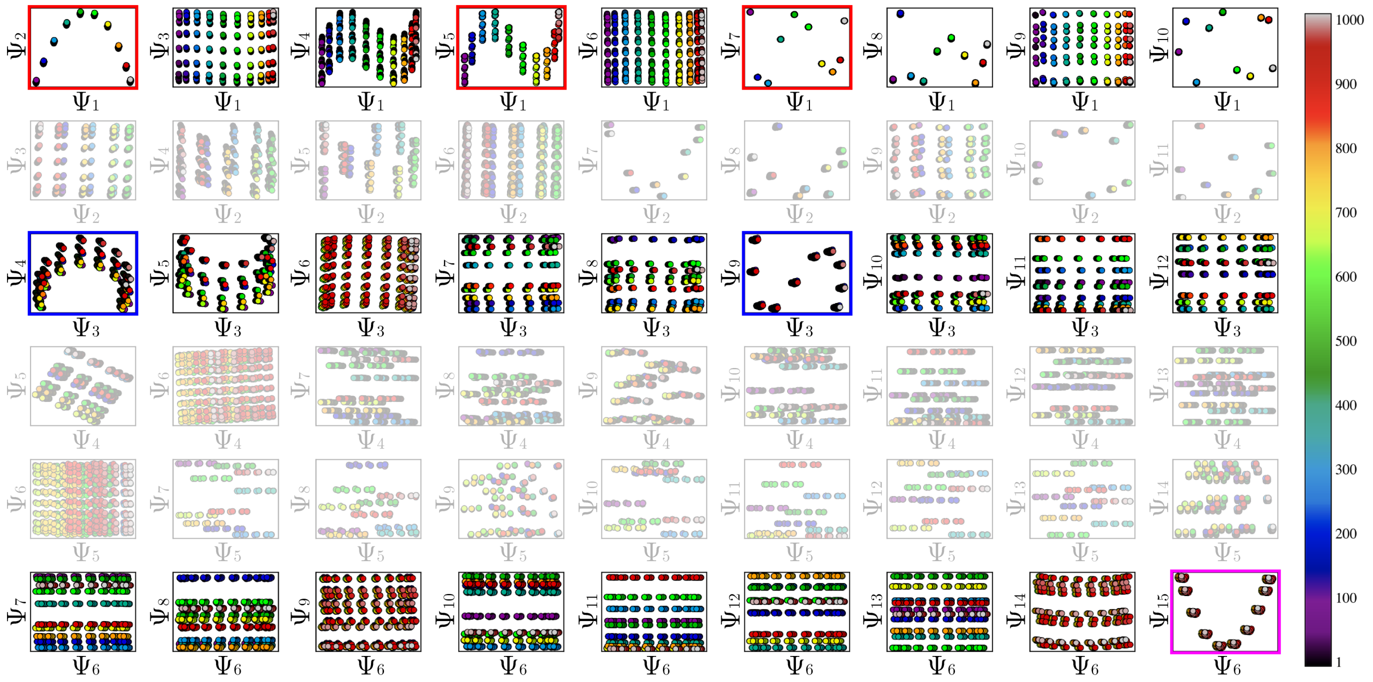

Fig. 15 A set of 2D subspaces projected from the embedding obtained from a PD in SS_3. Conformational modes are demarcated in red, blue and magenta boxes for CM_1, CM_2 and CM_3, respectively. To increase visibility of relevant patterns, the opacity of all other rows has been decreased.

C. Example of non-trivial boundary conditions due to steric hindrance

The initial 20×20 rectangular state space is displayed in Figure 16-A, where red boxes indicate states that were removed to form a grid with octagonal boundaries. The schematic in Figure 16-B provides some context for the possibility of a non-rectangular state space, which can be envisioned as a top-down view of (i) a large domain that opens and closes, and (ii) a small domain that translates left and right. Naturally, due to steric hindrance, while the larger domain is in a closed or half-closed state, the smaller domain is impeded from accessing a subset of its possible states, and vice versa. The eigenbasis obtained after application of a set of high-dimensional rotations[13] (of dimension $d = 15$) is shown in Figure 16-D. The required operators were estimated manually, and included several large and small transformations: $\{R_{5,6}(40°)$, $R_{2,6}(-15°)$, $R_{2,5}(3°)$, $R_{2,9}(4°)$, $R_{6,9}(20°)$, $R_{6,12}(-25°)$, $R_{2,11}(-6°)$, $R_{9,11}(25°)$, $R_{9,15}(5°)$, $R_{6,11}(3°)\}$.

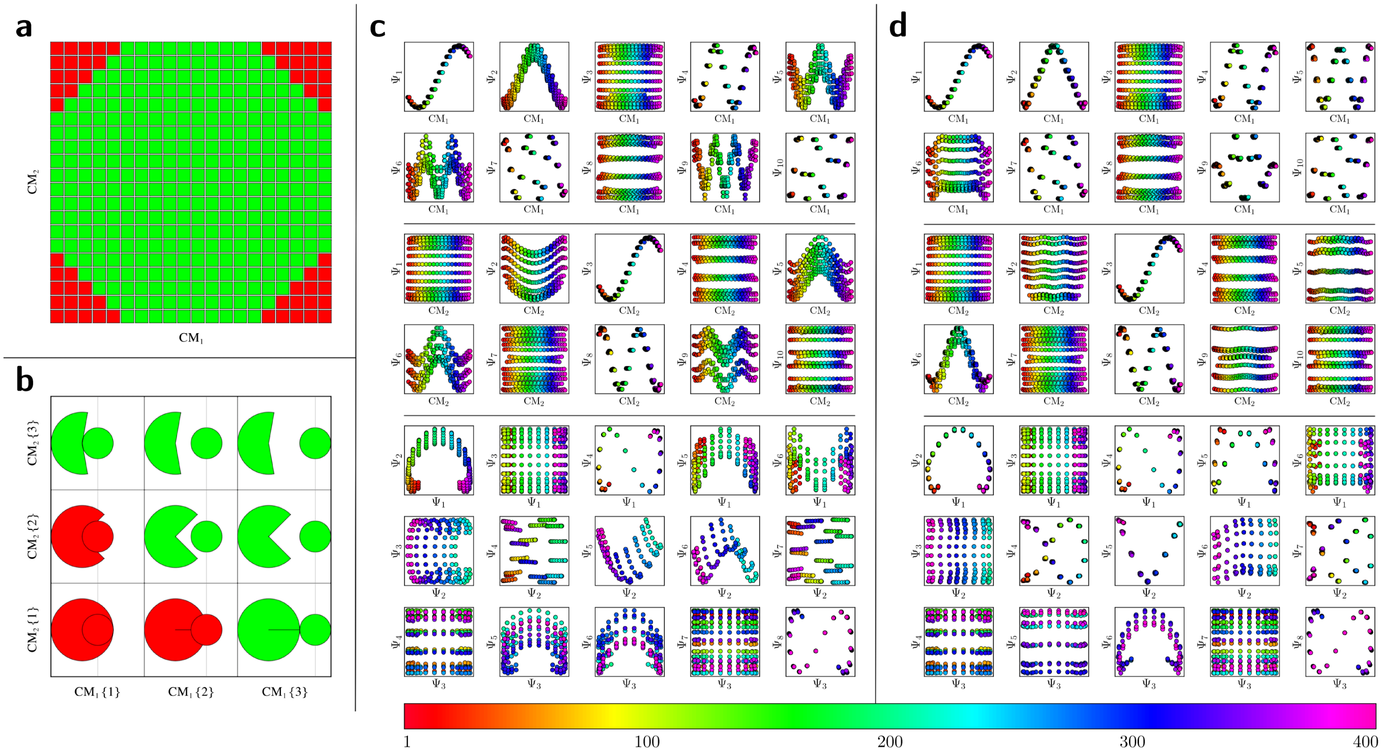

Figure 16. An example of non-trivial boundary conditions due to steric hindrance (**b**), with associated octagonal state space (**a**) of EDMs and corresponding eigenfunctions (**c**). In **d**, an ad-hoc eigenfunction realignment is applied to demonstrate innate features of the embedding.

Appendix. Description of symbols and abbreviations

Name	Description
CM	Conformational motion
n	Intrinsic dimensionality of the system, with one degree of freedom per CM
SS_n	State space with n degrees of freedom, where each state is a unique conformation of the system
M_n	Number of unique ground-truth states defining a given SS_n : $M_n \in \{20, 400, 1000\}$ for $n \in \{1, 2, 3\}$
Ω	Compact Riemannian n-dimensional manifold
ψ_k	Eigenfunctions of the Laplace-Beltrami operator
Ψ_i	DM eigenvector of the n-manifold
L	Lissajous curves; $L_{p,q} \in L$
T_k	Chebyshev polynomial of the first kind; e.g., $T_2(x) = 2x^2 - 1$ for the parabola
ε	Gaussian bandwidth used in DM Gaussian kernel
m	Number of atoms per atomic-coordinate structure (ACS)
V	Number of voxels per electron density map (EDM)
P	Number of pixels per image obtained from a projection direction (PD)
d	Dimension of orthogonal matrix O (and R_{ij}) applied on embedded space in $\mathbb{R}^d$
R_{ij}	2D rotation sub-matrix operating on $\{\Psi_i, \Psi_j\}$, of which there are $d(d-1)/2$

Supplementary notes and references

† The current section is a near replica of similar descriptions of DM and PCA provided in our companion article,[13] with the exception of a new subsection detailing the Laplace-Beltrami operator.

1 R. R. Coifman and S. Lafon, Diffusion maps, *Applied and Computational Harmonic Analysis*, 2006, **21**, 5–30.
2 A. L. Ferguson, A. Z. Panagiotopoulos, P. G. Debenedetti and I. G. Kevrekidis, Systematic determination of order parameters for chain dynamics using diffusion maps, *Proceedings of the National Academy of Sciences*, 2010, **107**, 13597–13602.
3 R. R. Coifman, Y. Shkolnisky, F. J. Sigworth and A. Singer, Graph Laplacian Tomography From Unknown Random Projections, *IEEE Transactions on Image Processing*, 2008, **17**, 1891–1899.
4 P. Grassberger and I. Procaccia, Measuring the strangeness of strange attractors, *Physica D: Nonlinear Phenomena*, 1983, **9**, 189–208.
5 P. Buser, *Geometry and Spectra of Compact Riemann Surfaces*, Springer, 1992.
6 J. Jost, *Geometry and Physics*, Springer-Verlag, Berlin Heidelberg, 2009.
7 D. S. Grebenkov and B.-T. Nguyen, Geometrical Structure of Laplacian Eigenfunctions, *SIAM Rev.*, 2013, **55**, 601–667.
8 D. Giannakis, P. Schwander and A. Ourmazd, The symmetries of image formation by scattering. I. Theoretical framework, *Opt. Express, OE*, 2012, **20**, 12799–12826.
9 P. Schwander, D. Giannakis, C. H. Yoon and A. Ourmazd, The symmetries of image formation by scattering. II. Applications, *Opt. Express, OE*, 2012, **20**, 12827–12849.
10 R. Feynman, R. Leighton and M. Sands, *The Feynman Lectures on Physics*, Chapter 12, Basic Books, New York, NY, 1965.
11 K. Pearson, On lines and planes of closest fit to systems of points in space, *Philosophical Magazine*, 1901, **2**, 559–572.
12 D. Lay, S. Lay and J. McDonald, *Linear Algebra and its Applications*, Pearson, 5th edn., 2016.
13 E. Seitz, F. Acosta-Reyes, S. Maji, P. Schwander and J. Frank, Recovery of Conformational Continuum From Single-Particle Cryo-EM Images: Optimization of ManifoldEM Informed by Ground Truth, *IEEE Transactions on Computational Imaging*, 2022, **8**, 462–478.
